# NBBt-test: a versatile method for differential analysis of multiple types of RNA-seq data

**DOI:** 10.1038/s41598-022-15762-x

**Published:** 2022-07-27

**Authors:** Yuan-De Tan, Chittibabu Guda

**Affiliations:** 1 Department of Genetics, Cell Biology and Anatomy, University of Nebraska Medical Center, Omaha, NE 68198 USA; 2 Center for Biomedical Informatics Research and Innovation (CBIRI), University of Nebraska Medical Center, Omaha, NE 68198 USA

**Keywords:** Computational biology and bioinformatics, Genetics

## Abstract

Rapid development of transcriptome sequencing technologies has resulted in a data revolution and emergence of new approaches to study transcriptomic regulation such as alternative splicing, alternative polyadenylation, CRISPR knockout screening in addition to the regular gene expression. A full characterization of the transcriptional landscape of different groups of cells or tissues holds enormous potential for both basic science as well as clinical applications. Although many methods have been developed in the realm of differential gene expression analysis, they all geared towards a particular type of sequencing data and failed to perform well when applied in different types of transcriptomic data. To fill this gap, we offer a negative beta binomial t-test (NBBt-test). NBBt-test provides multiple functions to perform differential analyses of alternative splicing, polyadenylation, CRISPR knockout screening, and gene expression datasets. Both real and large-scale simulation data show superior performance of NBBt-test with higher efficiency, and lower type I error rate and FDR to identify differential isoforms and differentially expressed genes and differential CRISPR knockout screening genes with different sample sizes when compared against the current very popular statistical methods. An R-package implementing NBBt-test is available for downloading from CRAN (https://CRAN.R-project.org/package=NBBttest).

## Introduction

It has been shown that alternative cleavage and polyadenylation (ACP) is a necessary step in the posttranscriptional processing and a versatile mechanism for posttranscriptional regulation of eukaryotic gene expression^1–3^. After transcription, a pre-mRNA is processed by capping at 5′ end, splicing, and cleaving in the 3′-untranslated region (3′UTR), which yields a new end for adding a polyadenylation (poly(A)) tail^3,4^. A poly(A) signal that is also referred to as poly(A) site is recognized and activated by a group of protein factors called polyadenylation factors^3–6^. Alternative poly(A) sites significantly increase the complexity of transcriptomes and proteomes. All poly(A) sites are in the terminal exon of a transcription unit giving rise to a tandem untranslated region (tandem UTR), which are exploited by the directed 3′ end sequencing methodologies. For a given transcription unit, transcript variants derived from the first poly(A) site (called poly(A) site 1) are assumed to have a transcript from the transcriptional start site (TSS) to poly(A) site 1. Similarly, transcript variants derived from poly(A) sites 2, 3, … are assumed to be derived from the TSS to poly(A) sites 2, 3, …, respectively. Therefore, within the same transcription unit, the transcript variants or isoforms have one-to-one correspondence to the location of poly(A) sites on the transcript.

Alternative splicing (AS) of RNA is an evolutionary mechanism in eukaryotes to produce multiple protein isoforms from a single gene^7^ to enhance the functional diversity of genes in a tissue-dependent and development-dependent manner^8^. Currently, AS events are observed in human, *D. melanogaster*, and *C. elegans*, respectively^9^. Misregulation of alternative splicing is associated with several diseases including cancers, where abnormal expressions or mutations in splicing factors are known to contribute to tumorigenesis^10^. AS possibly occurs in two non-neighboring exons (cassette) resulting in skipping exon, 3′UTR or 5′UTR splice sites, or in multiple exons, leading to multiple skipped exons or multiple-exon exclusion^11^. Intronic retention can result from mutations in splice sites or regulatory sequences.

CRISPR/Cas9 (clustered regularly interspaced short palindromic repeats/CRISPR associated protein 9) system has become a powerful tool for genome editing with many applications in the identification of cancer driver genes, drug-resistant genes, and others involved in metabolism in the mammalian cells^12–16^. This system is constructed with a single-guide RNA (sgRNA) of a short nucleotide sequence complementary to a targeted DNA sequence region of a selected gene and Cas9 nucleases inducing double-strand break (DSB) in this region. When a DSB is repaired by Cas9 in a non-homologous end-joining (NHEJ) way, a repairing error occurs with a high probability due to an insertion/deletion mutation that is likely to cause a codon frameshift, which leads to a premature stop codon^17^. As a result, a targeted locus would be efficiently knocked out. A recently developed lentiviral delivery method has made it possible to create large-scale genome-wide CRISPR/Cas9 knockout libraries targeting ~ 10^4^ genes^18^. These libraries allow both negative and positive selection screens to be conducted in mammalian cell lines in a cost-effective manner^16,18–21^. All genome-wide CRISPR screens use cell growth as a phenotypic measure. Either a screen is referred to as positive selection screen in which gene knockout results in a selective advantage for cells such as drug or toxin resistance or a screen is a negative selection screen in which a gene knockout results in a selective disadvantage in that cell such as decreased proliferation in cancer cells.

In CRISPR/Cas9 knockout screens, genes targeted by set of multiple sgRNAs constitute a mutant pool. Cells that carry sgRNA targeting genes resisting to strong selection pressure would be enriched and the signals (RNA) becomes strong so that they are easily detected, while signals from negative selection are weak because of depletion during screen. So, a lot of genes including those promoting cell growths and housekeeping genes are negatively selected in screen. Detection of the genes targeted by sgRNAs and targeting sites could be resolved by high-throughput sequencing using libraries.

Therefore, ACP events, AS events and sgRNAs are all detectable by sequencing the transcriptome using RNA-seq where the short sequencing reads derived from different structural elements of genes can be mapped to corresponding regions and annotated using a given reference genome. There are many methods for differential expression analysis of RNA-seq data at the gene level, most of which use similar information across gene expression profiles to estimate dispersion^22–24^. For example, popular methods such as edgeR^23–25^ and DESeq2^22^ model estimate of dispersion for each gene by using a common estimate across all genes with similar expression strength as a weighted conditional likelihood. The first version of DESeq^26^ determines dispersion estimates by modeling the dependence of the dispersion on the average expression value over all samples. BBSeq^27^ models the dispersion on the mean with absolute deviation of dispersion estimates by excluding the influence of outliers. DSS^28^ is a Bayesian approach to estimate the expression dispersion of genes that accounts for the heterogeneity of dispersion values for different genes. As an improvement to DESeq, DESeq2 uses shrinkage estimation for dispersions and fold changes to improve the stability and interpretability of estimates^22^. On the other hand, ShrinkBayes^29^ and baySeq^30^ estimate priors using a Bayesian model over all genes, and then provide posterior probabilities for differential expression. While these methods are suitable for determining gene-level expression profiles, they have severe limitations when used for differential splicing, differential polyadenylation, and differential sgRNA target analysis because RNA-seq count data for ACP, AS and CRISPR screens do not provide gene-level dispersion and their dispersion estimates are not accurate at sub-gene level. For this reason, a variety of methods exclusively for detecting differential splicing events have been developed. These methods fall under count-based methods and isoform resolution methods. Count-based methods include DEXSeq^31^, DSGseq^32^, SplicingCompass^33^, rMATS^34,35^, rDiff-parametric, and SeqGSEA^36^, while Cufflink^37^and DiffSplice^38^ are examples of isoform resolution methods. For identifying differential CRISPR knockout screen, model-based analysis of gene-wide CRISPR/Cas9 knockout (MAGeCK)^18^ has recently been developed. MAGeCK performs at either sgRNA target level or gene level. The other algorithms such as RNAi gene enrichment ranking (RIGER)^39^, redundant siRNA activity (RSA)^40^, and permutation-based non-parametric analysis (PBNP)^17^ can be used to identify differential hits at gene level.

In summary, the current methods use a common dispersion estimator from the gene expression profile to model the dispersion estimate of each gene in RNA-seq data, which is not an ideal strategy because the range of gene expression is large across the genome and the gene expression profiles widely vary among different type of transcriptome measurement experiments. Hence the use of a common dispersion estimator strongly impacts the estimates of individual dispersions of genes, which affects the stability and interpretability of estimates. In addition, most of the current methods do not really consider the effects of small samples on differential analysis and we found from our heatmap analysis that many differentially expressed genes or isoforms detected by these statistical methods have larger null variation than biological variation. To address these issues, we here develop a novel statistical framework to do differential analysis of multiple types of RNA-seq experimental data. This new method named as negative beta binomial t-test (NBBt-test) expands the prior approaches of Beggerly et al.^41^ and Tan et al^42^. NBBt-test is a versatile method for identifying differential splicing events, differential adenylation events, differential CRISPR knockout screens and differentially expressed genes. This methodology incorporates t-test, fold-change and F-test so that it works well for both small and large sample sized datasets. We compared NBBt-test with the existing statistical methods using both experimental and large-scale simulated RNA-seq and CRISPR FACS datasets and found that our methodology is very robust with very low type I error rate or FDR, high power, high efficiency. An R-package implementing NBBt-test is available for downloading from CRAN (https://CRAN.R-project.org/package=NBBttest).

## Results

### Inflation-shrinkage variable

To address variance inflation and shrinkage issues raised in small-sample experiments, we here offer inflation-shrinkage variable (*ρ*) to express statistical effects of small samples. $\rho_{gi}$ is defined as geometric mean of $\varphi_{gi}$ and $\zeta_{gi}$^42^:

1 $$\rho_{gi} = \sqrt {\zeta_{gi} \varphi_{gi} } .$$

To avoid infinity due to zero count, we modify $\varphi_{gi}$ as

2 $$\varphi_{gi} = \max \left[ {\frac{{\min \left( {{\text{X}}_{{{gAi}}} } \right) + 1}}{{\max \left( {{\text{X}}_{{{gBi}}} } \right) + 1}},\frac{{\min \left( {{\text{X}}_{{{gBi}}} } \right) + 1}}{{\max \left( {{\text{X}}_{{{gAi}}} } \right) + 1}}} \right]$$

$$X_{gAi}={x_{gAi1} ,..., x_{gAin_{A}}} $$ and $$X_{gBi}={x_{gBi1} ,..., x_{gBin_{B}}} $$. We also modify $$\zeta_{gi}$$ as

3 $$\zeta_{gi} = \ln \left( {\frac{{\overline{X}_{gi} \sigma_{gi}^{2} + 1}}{{\overline{X}_{Agi} \sigma_{Agi}^{2} + \overline{X}_{Bgi} \sigma_{Bgi}^{2} + 1}}} \right)$$

$\varphi_{gi}$ is used to measure overlap between datasets A and B for sgRNA *i* targeting gene *g* or isoform *i* resulted from differentially splicing or adenylating within gene *g* and $\zeta_{gi}$ is used to measure homogeneity or dispersion of data within conditions where $\overline{X}_{gi}={\frac{1}{2}\overline{X} }_{gAi}$, $\overline{X}_{gAi} = \frac{1}{{m}_{A} } {\mathrm{S}}_{gAij}$ and $S_{gAi}=\sum\nolimits_{j=1}^{{m}_{A}}{x_{gAij}}$ where ${x}_{gAij}$ is the count of sgRNA *i* within gene *g* in sample *j* in condition A, ${S}_{gAi}$ is the sum of count of sgRNA *i* in condition A, ${\overline{X} }_{gAi}$ and ${\overline{X} }_{gBi}$ are, respectively, means of sgRNA *i* within gene *g* in conditions A and B. For the count data of RNA reads, $x_{gAij} \ge 0$ and $x_{gBij} \ge 0$. If $\overline{X}_{gAi} = \overline{X}_{gBi} = 0$, then $\overline{X}_{gi} = 0$ and $\zeta_{gi} = 0$. Both $\varphi_{gi}$ and $\zeta_{gi}$ are defined in [$0,\infty$]. For a given two-condition experiment, Eq. (2) indicates that if $X_{gAi} = \{ x_{gAi1} , \ldots ,x_{{gAin_{A} }} \}$ and $X_{gBi} = \{ x_{gBi1} ,\ldots ,x_{{gBin_{B} }} \}$ do not overlap, then $\varphi_{gi} > 1$, otherwise, $\varphi_{gi} < 1$. $\zeta_{gi} < 1$ defines large within-condition variation or noise while $\zeta_{gi} > 1$ implicates that the noises are small, and observations are relatively consistent across replicates within conditions. $\varphi_{gi}$ and $\zeta_{gi}$ are independent variables because $\varphi_{gi}$ depends on maximum and minimum values of two datasets, while $\zeta_{gi}$ is determined by means and variances of these two datasets, maximum and minimum values are independent of means and variances. Hence, a measure for both data gap between two conditions and small null variation can be measured by $\rho_{gi}^{2} = \varphi_{gi} \zeta_{gi}$, that is, if $\varphi_{gi} > 1$ and $\zeta_{gi} > 1$, then $\rho_{gi}^{2} > 1$. For example, ${X}_{A}$= {4764, 4602, 4538} and ${X}_{B}$= {7877, 7524, 7871} have gap and good homogeneity (small dispersion within conditions). Our calculation shows $\varphi = 1.579 > 1$ and $\zeta = 1.691$, and $\rho = 1.633$, indicating that data are relatively consistent across all replicates within conditions, which is well agreeable with the observations. Another example is ${X}_{A}$= {1390, 1482, 1561} and ${X}_{B}$= {1540, 1270, 1217}. One can see that these two datasets overlap but also have poor homogeneity (large noises). Our calculation shows $\varphi = 0.902 < 1$, $\zeta = 0.194 < < 1$ and hence $\rho = 0.418 < 1$, which is again agreeable with the observations. Equation (2) shows that $\varphi_{gi}$ is similar to fold change and from Eq. (3), $\zeta_{gi}$ is similar to F-statistic.

### Model and estimation of parameters

Different from DESeq2^22^, NBBt-test begins with a count sub-matrix with ${n}_{g}$ isoforms of gene *g* for rows and ${m}_{k}$ replicates in condition *k* for columns. The matrix entries indicate the number of sequencing reads that have been unambiguously mapped to a gene. For the sake of convenience, we begin with CRISPR read count data. Suppose we select G genes of interest. Gene *g* (*g* = 1, …, *G*) has $n_{g}$ sgRNAs to hit a DNA sequence in a screen experiment. Let $x_{gij}$ be a normalized or adjusted count (e.g., RPKM (Reads Per Kilobase Million) or FPKM (Fragments Per Kilobase Million) or TPM (Transcripts Per Kilobase Million)) of RNA reads within gene *g* targeted by sgRNA *i* (*i* = 1, …, $n_{g}$) in experiment (biological replicate experiment) *j* (*j* = 1, …, ${m}_{k}$) in condition k*.* For differential analysis, we here just consider two conditions such as treatment and control groups, so *k* = 1, 2. For the convenience, our NBBt-test is restricted to sgRNAs targeting a gene, in other words, count data of multiple RNA reads within a gene are used as a sub-matrix with rows for RNA within gene g targeted by a sgRNA and columns for samples or replicates. NBBt-test first works with a sub-matrix and *iterates G sub-matrices*. Similar to DESeq2^22^, NBBt-test also assumes that the count of RNA reads follows a negative binomial distribution with a specific number r of failures to RNA sequencing reads in a RNA species and probability *p* of this RNA species (also called RNA isoform) to be sequenced. For the count data of CRIPSR knockout screen, *p* is estimated by proportion of sequencing RNA sequences from gene *g* targeted by a sgRNA*.* Different from DESeq2, we are interested in *p* instead of r. We here use $p_{gij} = x_{gij} /X_{g}$ to initially estimate the proportion where $X_{g}$ is the largest total count over all ${n}_{g}$ sgRNAs among $m$ replicate experiments. Using $X_{g}$ instead of ${X}_{gj}$ to calculate $p_{gij}$ is because a set of sgRNAs are already designed before the experiment and hence difference among replicate experiments results from technical noise instead of biological system error. Using ${X}_{gj}$ to calculate $p_{gij}$ would increase proportion of technical noise due to the fact that ${X}_{gj}$ contains noise among $m$ replicates. Our simulation also shows that ${p}_{gij}=\frac{{x}_{gij}}{{X}_{g}}$ is better than ${p}_{gij}=\frac{{x}_{gij}}{{X}_{gj}}$ (results not shown). In the negative binomial distribution,$p_{gij}$ follows a beta distribution^43^: $p\sim Beta\; (\alpha ,\beta )$. Mean and variance of the proportion for a sgRNA targeting a gene are given by parameters $\alpha$ and $\beta$^41^. To consider the case that RNA experimental sample sizes are limited, we use weights to optimally estimate parameters across replicates^41,42^ (see Supplementary Statistical methods for detail). Due to $\sum\nolimits_{j = 1}^{m} {w_{gj} = 1}$, sum of weighted means over all replicates in a condition is expectation of the mean. With the weights, the proportion of sgRNA *i* targeting gene *g* in a condition is estimated by $\hat{p}_{gi} = \sum\nolimits_{j = 1}^{m} {w_{gj} \hat{p}_{gij} }$ and the variance is also unbiasedly estimated using weights^41,42^ (see Supplementary Statistical methods for detail). For poly(A) RNA-seq or splicing/exon RNA-seq, since RNA in an experiment is abstracted from a library, different libraries would have different total RNA amounts, that is, RNA amount is fixed by library size. To remove difference due to library sizes, ${p}_{gij}$ is defined as ${p}_{gij}=\frac{{x}_{gij}}{{X}_{gj}}$ where ${X}_{gj}=\sum_{i=1}^{{n}_{g}}{x}_{gij}.$

Since we have weights for parameters ($\alpha , \beta$, *p*, and *V*), we use an iteration algorithm to optimally estimate these parameters driven by estimating weights. However, this iteration algorithm is not sensitive to count size, in all RNA-seq data many RNA isoforms or sgRNAs have small read counts. Small counts would result in high similarity of proportions among few replicates, which leads variances to be much smaller than differences between means so that the t-statistics are inflated. To avoid occurrence of this phenomenon, we propose another alternative estimate of *p* variance:

4 $$V_{gi} = \frac{1}{X}_{g} \left[ {\frac{{1 + X_{gi} }}{{X_{g} }}\left( {1 - \frac{{1 + X_{gi} }}{{X_{g} }}} \right)} \right]$$

where $X_{gi} = \sum\nolimits_{j = 1}^{m} {x_{gij} }$ and $X_{g} = \sum\nolimits_{i = 1}^{{n_{g} }} {X_{gi} }$. The variance for proportion (p) of sgRNA *i* targeting gene g or RNA isoform *i* within gene g would be given by choosing a bigger one from these two variances estimated by iteration algorithm (see Supplementary Statistical methods for detail) and by Eq. (4). Equation (4) shows that the lower bound of $\hat{V}_{gi}$ is $\frac{1}{{X_{g}^{2} }}\left( {1 - \frac{1}{{X_{g} }}} \right)$ > 0 when $X_{gi} = 0$.

### T-test for differential expression of RNA isoforms or sgRNAs

With $\hat{p}_{Agi}$, $\hat{p}_{Bgi}$, $\hat{V}_{Agi}$, $\hat{V}_{Bgi}$, and $\rho$ given in conditions A and B, a new t-statistic is defined as $t_{gi}^{\alpha } = \frac{{\rho_{g} }}{{\omega_{\alpha } }}t_{gi}$ for differential screens of the *i*th sgRNA targeting gene g or $t_{gi}^{\alpha } = \frac{{\rho_{gi} }}{{\omega_{\alpha } }}t_{gi}$ for differential expression of the *i*th RNA isoform of gene g where $t_{gi}$ is t-statistic of Baggerly et al. (see Supplementary Statistical methods for details), $\rho_{g}$ is gene-wise inflation-shrinkage variable at gene level and $\rho_{gi}$ is isoform-wise inflation-shrinkage variable at RNA isoform level. $\omega_{\alpha }$ is a null $\rho$ given by simulation under significance level of $\alpha$ (see Supplementary statistical methods for details). Different from variance and fold change shrinkages of DESeq2, $\rho /\omega_{\alpha }$ has inflation or shrinkage function. $\omega_{\alpha }$ is due to false positive findings under significance level of $\alpha$ by multiple null simulations and hence is used as a threshold for an observed $\rho$. This means that $t_{gi}^{\alpha }$ would be inflated ($t_{gi}^{\alpha } > t_{gi}$) when $\rho > \omega_{\alpha }$, $t_{gi}^{\alpha }$ would be shrunken ($t_{gi}^{\alpha } < t_{gi}$) when $\rho < \omega_{\alpha }$ and $t_{gi}^{\alpha } = t_{gi}$ when $\rho = \omega_{\alpha }$. In practice, when two datasets have a gap and are consistent across replicates, we have $\rho > \omega_{\alpha }$, however, if an isoform or a sgRNA has outlier and overlapped datasets or big noises, then $\rho < \omega_{\alpha }$. Only in the small-sample experiments, the first case suggests that in probability these two datasets more possibly come from different distributions than from a distribution while in the second case, the two datasets are more likely sampled from a distribution. Hence, in most cases, true differences between conditions would be inflated so that true positives would be found and the differences between two conditions due to noises would be strongly shrunken to be very small so that false positive findings would be excluded.

Although CRISPR sgRNAs and RNA isoforms occur at sub-gene level, a set of CRISPR sgRNAs targeting a set of DNA sequences of a gene was already designed before experiment, the variation of RNA reads of sgRNAs is fixed or due to a fixed effect, while the variation of isoform RNA reads is uncertain, depends on gene structure such as alternative poly(A) sites, alternative splicing sites in UTR, exons, introns, in other words, due to the random effect. This factor has not been considered in the current methods. To use the information on this factor, we introduce gene-wise $\rho_{g}$ to adjust the difference in sgRNA counts targeting a gene between conditions A and B. RNA isoforms are derived from alternative splicing sites or alternative polyadenylation sites, hence, amounts of RNA isoforms are uncertain at sub-gene and gene levels, or depend on response of the gene to conditional effect, that is to say, variation of an RNA isoform is not fixed at either splicing sites or poly(A) sites and at gene level but due to random effect. For this reason, we introduce isoform-wise $\rho_{gi}$ to adjust difference between two conditions.

### T-test for differential expression of genes

To test for differential expression of genes or differential screen of genes targeted by a set of sgRNAs is the main purpose of NBBt-test. For doing so, we use sum of the read counts over all the designed sgRNAs targeting the DNA sequence of a gene as the read count of this gene. For CRISPR and RNA isoform data, we use the proportion ($p_{gj}$) of read count of gene *g* in replicate experiment* j* to the total read count across all genes in a condition as initial estimate of parameter *p* of a negative binomial distribution: ${p}_{gj}={X}_{gj}/{X}_{j}$ where $X_{gj} = \sum\nolimits_{i = 1}^{{n_{g} }} {x_{gij} }$ and $X_{j} = \sum\nolimits_{g = 1}^{G} {X_{gj} }$($j=1, 2, \dots , m, g=1,2,\ldots , G$). After normalizing the data, the total count over all genes is the same for all replicates, that is, ${X}_{11}=\cdots={X}_{1{m}_{1}}={X}_{21}=\cdots={X}_{2{m}_{2}}=X$. For normalized RNA-seq data without RNA isoforms, $X_{gj}$ is count of RNA reads for gene g in replicate j. We still assume that $p_{gj}$ follows beta distribution with alpha ($\alpha$) and beta ($\beta )$. Therefore, we use the iteration algorithm with weights (see Supplementary statistical methods) to estimate $p_{g}$ and $V_{g}$ in a condition. With the estimated parameters, a new t-statistic for differential expression or screen of gene *g* is defined as $t_{g}^{\alpha } = \frac{{\rho_{g} }}{{\omega_{\alpha } }}t_{g}$ where $t_{g}$ is t-statistic of Baggerly et al. (see Supplementary statistical methods for detail),$\rho_{g}$ is gene-wise inflation-shrinkage variable and $\omega_{\alpha }$ is a threshold for $\rho_{g}$. Therefore, at gene level, $t_{g}^{\alpha }$ is inflated with $\rho_{g}$ > $\omega_{\alpha }$ or shrunken with $\rho_{g}$ < $\omega_{\alpha }$.

### Estimation of omega ($\omega_{\alpha }$)

In new t-test definitions and in Eqs. (S27) and (S38) in Supplementary Statistical Methods, $\omega_{\alpha }$ is a null $\rho$-value detected at significance level of $\alpha$, used as a threshold of $\rho_{gi}$. The $\omega_{\alpha }$ can be estimated by using null data by following the steps:

*Step 1:* Perform simulation using negative binomial distributions with parameters given by inputting dataset to produce a null count dataset consisting of the same sgRNAs or RNA isoforms and the same number of replicates in each condition with the original real dataset.

*Step 2:* Given significance level of $\alpha$, perform beta t-test of Beggerly et al.^41^ on the simulated null dataset by setting $\rho = 1$ and ${\omega }_{\alpha }$=1, and calculate new $\rho$ values using Eqs. (1–3).

*Step 3:* Sort all *p*-values from largest to smallest ($p_{1} > , \cdots , > p_{i^{\prime}} > , \cdots , > p_{S}$) where *i*′ is in the order sequence of S *p*-values ($S = \sum\nolimits_{g}^{G} {n_{g} }$, *g* = 1, …, G), select sgRNAs with ${p}_{{i}^{{\prime}}\ge j}<\alpha$, and calculate $\rho$ values ($\rho_{j} , \ldots ,\rho_{S^{\prime}}$) of these sgRNAs selected using the simulated null data. *K* = *S–j* sgRNAs are assumed to have *p*-values < $\alpha$.

*Step 4:* Sort the *K* $\rho$ values from smallest to largest ($\rho_{1} < \rho_{2} , \cdots , < \rho_{k^{\prime}} < , \cdots ,\rho_{K - 1} < \rho_{K}$) and choose a $\rho$ value at $\frac{{k}^{{\prime}}}{K}\ge 0.85$ : $\rho \left(\alpha \right)={\rho }_{{k}^{{\prime}}}$ where *k*′ is in the order sequence of *K* $\rho$ values. $\frac{{k}^{{\prime}}}{K}\ge 0.85$ suggests that at least 85% false positives selected at significance level of $\alpha$ would be controlled. However, if K < 7, only *K*′=*K* has *K*′/*K* ≥ 0.85. In this case, we choose mean over K $\rho$ values:$\rho \left(\alpha \right)=\frac{1}{K}({\sum }_{\mathrm{k}=1}^{\mathrm{K}}{\rho }_{k})$. Another case that no null RNA species has *p* value < $\alpha$ also possibly occurs. In this case, we choose the largest $\rho$ value among all K RNA species: $\rho \left(\alpha \right)={max}_{\mathrm{k}=1}^{\mathrm{K}}\left({\rho }_{k}\right)$.

*Step 5:* repeat from step 1 to step 4 for *s* times and average $\rho$ values over *s* simulated null datasets: $\omega_{\alpha } = \frac{1}{s}\sum\nolimits_{r = 1}^{s} {\rho_{r} } (\alpha ).$

**Figure 1:**
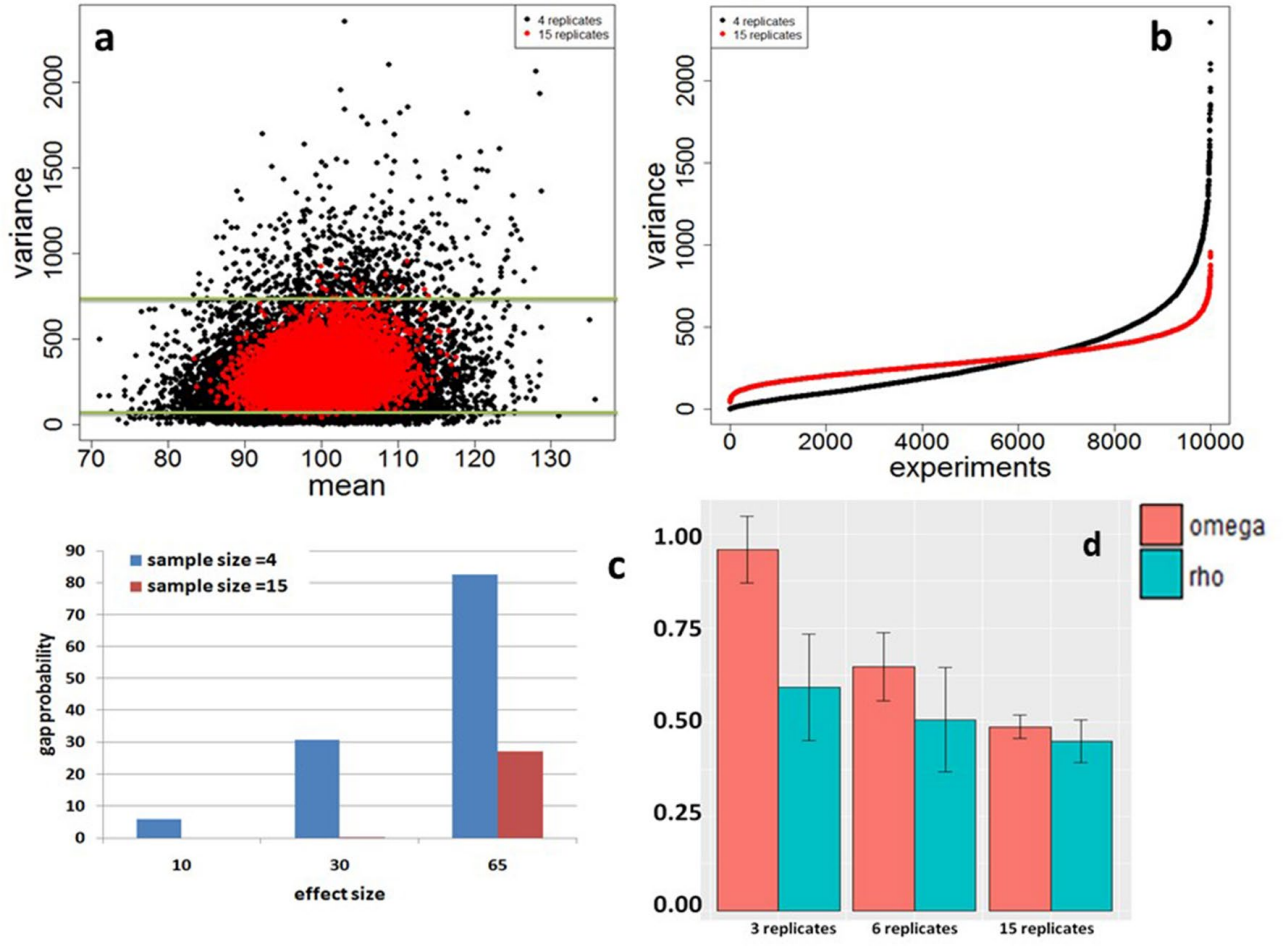
Effects of small samples. Experimental data of 10,000 genes were respectively simulated with equal sample size = 4 and 15 from a negative binomal distribution NB (100, 50). (**a**) Variance distributions along mean where many variances of samples with equal sample size = 4 under green line are smaller than those with equal sample size = 15 and some variances of samples with 4 replicates per group above blue line are larger than those with equal sample size = 15 replicates per group. (**b**) Sorted variances distributions along experiments. (**c**) Four sets of two-sample data of 10,000 genes were randomly sampled with equal sample sizes of 4 and 15, effect size E = 10, 30, and 65 from normal distribution NB (100, 50) or NB(100+E, 50) with equal probability. (**d**) impact of sample size on $\rho \;{\text{and estimate of}}\; \omega$.

To verify the estimate of $\omega_{\alpha }$**,** we simulated null expression data of 10,000 genes in two conditions in three cases: each condition has 3, 6 and 15 replicates and calculated $\rho$ and applied the above estimation method to estimate $\omega_{\alpha }$. The result is shown in Fig. 1d. Our simulation result shows that in the case of 3 replicates, $\omega_{\alpha }$ is 0.35 larger than $\rho$, in the case of 6 replicates, $\omega_{\alpha }$ is 0.15 larger than $\rho$, while when the sample size = 15, $\omega_{\alpha }$ is only 0.05 larger than $\rho$. This result is expected because the smaller the sample size, the larger the probability of gap occuring between two samples and the larger the $\omega_{\alpha }$. When sample size is larger than or equal to 15, probability of gap occurring between two samples is zero when effect size is less than or equal to 30 (Fig. 1c).

The number of iterations of the simulation depends on the number of RNA species and $\alpha$. This is akin to controlling false discovery rate because number of *p*-values < $\alpha$ is determined by gene number and $\alpha$. For example, 100 genes would have 5 false positives with 5 $\rho$ values expected by $\alpha$ = 0.05, 1000 genes would have 50 false positives with 50 $\rho$ values expected by $\alpha$ = 0.05 and 10,000 genes would produce 500 false positives with 500 $\rho$ values expected by $\alpha$ = 0.05. In this sense, we expect that we will exclude 85% of 500 false positives detected at level of $\alpha$ in 10,000 genes so $\omega_{\alpha }$ would greatly control the false discovery rate.

### Statistical effects of small samples

In small-sample experiments, data tend to have either smaller or larger null variances along with means (Fig. 1a). For example, in a dataset of 10,000 experiments with two conditions randomly sampled from a negative binomial distribution NB(100, 50), 70% of the experiments with 4 replicates (n = 4) per sample had smaller variances than those with 15 replicates (n = 15) per sample and 30% of experiments with 4 replicates per sample had much larger variances than those in two samples with 15 replicates per sample (Fig. 1b). If mean-distances between two samples are the same or approximate in these two cases, then 70% of t-statistics would be inflated due to small standard errors and 30% would be significantly shrunken by larger standard errors. The t-statistic inflation phenomenon has been noticed by Baldi and Long^44^, Tusher et al^45^, Cui and Churchill^46^ in high-throughput data. Another statistical effect of small samples is that there is a big chance in high-throughput experiments that a data gap event occurs between experimental conditions. For example, random sampling with equal sample size n = 4 and effect size E = 65 from NB(100,50) or NB(100+E, 50) with equal probability for each of 10,000 experiments showed that 82.57% of experiments have a data gap between two conditions (Fig. 1c), while drawn with equal sample size n = 15, the gap probability was significantly reduced to 27.02% (Fig. 1c). The probability decreases with decrement of effect size E. For instance, with effect size E = 30, the experiments with 4 and 15 replicates per group had gap probabilities of 30.63% and 0.23%, respectively (Fig. 1c). When effect size E = 10, the experiments with 4 and 15 replicates per sample had gap probabilities of 5.83% and 0%, respectively.

### Comparative benchmarks

We used both simulated and real RNA-seq datasets to test the performance of NBBt-test for differential analysis in comparison to the current popular methods such as edgeR^23,24^, DESeq2^22^, DEXSeq^31^, and MAGeCK^18^. Love et al^22^ compared DESeq2 to DSS^28^, EBSeq, voom^47^ and the SAMseq method of the samr package^48^. We therefore did not select DSS, EBSeq, voom and SMseq to compare NBBt-test. For all the selected methods, *p*-values from genes or isoforms or sgRNAs with non-zero read counts across samples were adjusted using the Benjamini–Hochberg procedure ^49^.

### Differential polyadenylation RNA isoforms

**Figure 2:**
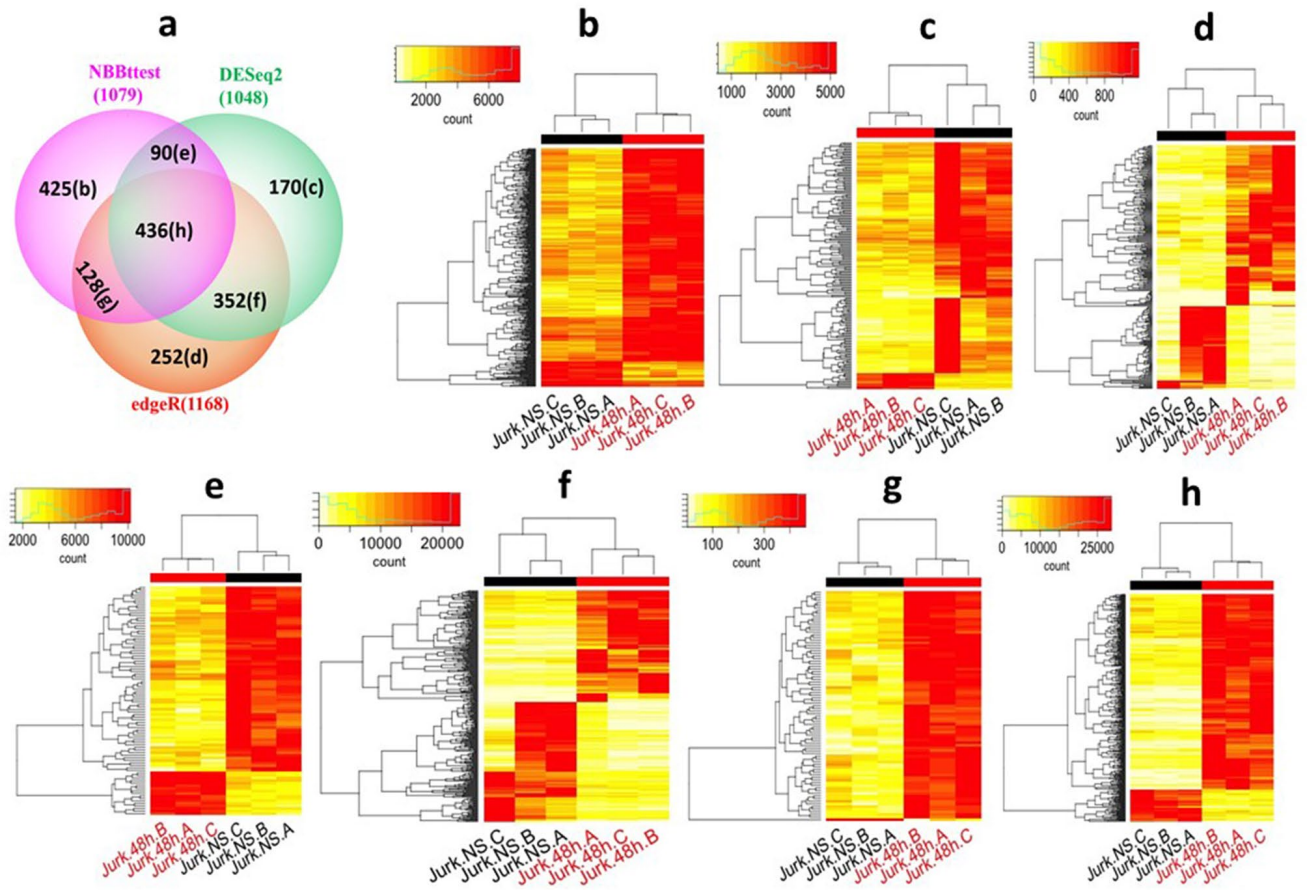
Venn-diagram and heatmaps of differential expressions of mRNA isoforms targeted poly(A) RNAs identified by different statistical methods. Venn-diagram: Venn-diagram (**a**) of poly(A) isoforms differentially expressed (DE) between control (no stimulation, NS) and treatment (stimulation 48 h) found by statistical methods edgeR, DESeq2, and NBBt-test. Heatmaps: Heatmaps (**b**–**h**) of poly(A) isoforms in each Venn-diagram cell were made by using antilog2 to inverse target expression value (x) to the original expression value (y): y = 2^×^ and normalizing isoform-wise expression value (y) across all replicates: ${n}_{ij}={y}_{ij}\frac{max(y)}{\max({y}_{i.})}$ where max(y) is maximum among all isoforms and all replicates of two groups and $\max({y}_{i.})$ is maximum among all replicates of two groups in isoform *i*. Value $n$ has the same order of magnitude in all isoforms in a given Venn-diagram cell. (**b**) Heatmap of 425 DE isoforms identified by NBBt-test only. (**c**) Heatmap of 170 DE isoforms identified by DESeq2 only. (**d**) Heatmap of 252 DE isoforms identified by edgeR only. (**e**) Heatmap of 90 DE isoforms identified by NBBt-test and DESeq2 only. (**f**) Heatmap of 352 DE isoforms identified by edgeR and DESeq2 only. (**g**) Heatmap of 128 DE isoforms identified by NBBt-test and edgeR only. (**h**) Heatmap of 436 DE isoforms identified by all three methods.

We used the count data of polyadenylated (Poly(A)) RNA reads^3,4^ from Jurkat T-cells of mouse non-stimulated (NS) and stimulated (48 h) with plate-bound antibodies to perform DESeq2, edgeR, and NBBt-test and used a Venn diagram to compare the findings of these methods(Fig. 2a). The differentially expressed poly(A) isoforms detected by NBBt-test show highly consistent expression across all replicates within each condition and distinct differences between the conditions (Fig. 2b,e,g and h), that is, Figs. 2b,e,g and h in heatmaps clearly show that differences in expression of all isoforms detected by NBBt-test between conditions 48 h stimulation and NS are definitely larger than those within conditions, while those detected by DESeq2 only (Fig. 2c), edgeR only (Fig. 2d), and commonly by both DESeq2 and edgeR (Fig. 2f) were not consistent across replicates within a condition because the differences in expression of many isoforms within conditions are larger than those between 48 h stimulation and NS.

### Validation of qPCR experiments

To validate that NBBt-test indeed performs better than DESeq2 and edgeR in identifying differentially expressed (DE) poly(A) RNA isoforms, we used qPCR results from the previous study^42^ to verify the predicted results obtained by applying these three methods to poly(A) RNA isoform data of genes TESK2, BC11B, UBL3, MST123, CD47, and KIAA0465. Gene TESK2 is a housekeeping gene with a single poly(A) site and the difference ${\Delta \Delta }Ct$ between cell stimulation and rest statuses was not significant at $\alpha <0.05$. Except for gene BC11B, the poly(A) RNA isoforms and qPCR result of all the other genes had the same expression directions (up- or down-expression) (Table 1). Gene UBL3 has three poly(A) sites and was detected by qPCR to have differential expression (${\Delta \Delta }Ct=0.54)$. DESeq2 did not find DE isoforms at all three poly(A) sites using RNA-seq data, while edgeR and NBBt-test identified DE isoform at the third site (30,338,534 bp), therefore, the differential expression value of gene BC11B was due to differential expression of isoform at this poly(A) site. Gene MST123 also has three poly(A) RNA isoforms and was detected by qPCR to have stronger differential expression (${\Delta \Delta }Ct$ = 2.42). DESeq2 found DE isoform at the first poly(A) site 38,270,255 bp, NBBt-test identified the DE isoforms at the first and second poly(A) sites (38,270,255 bp and 38,270,363 bp), while edgeR detected DE isoforms at all these three sites. At the third poly(A) site, both NBBt-test and DESeq2 did not find DE isoform, hence it did not contribute to differential expression of the gene. Thus, stronger DE value of MST123 is likely attributed to differential expression at the first two poly(A) sites. Gene CD47 has two poly(A) sites. DESeq2 and NBBt-test found that these two isoforms were differentially expressed between cell stimulation and rest statuses, but edgeR did not find them. Therefore, differential expression of CD47 might be attributed to differential expression of these two isoforms. In gene KIAA0465, all the three methods found the first isoform differentially expressed. For the housekeeping gene, TESK2, both edgeR and DESeq2 identified differential expression while the NBBt-test did not find it. If a gene has significant qPCR difference (ΔΔCt) between cell stimulation and rest statuses under $\alpha$ = 0.05 and an RNA isoform within this gene is found to be differentially expressed by two or all these three methods under FDR = 0.05, then this isoform is determined to be very possibly truly differentially expressed, otherwise, DE of this isoform is false. Using this way, true DE isoforms were inferred and listed in the last column of Table 1. Compared findings of a statistical method to true DE isoforms in the last column in Table 1, we found that the ratio of true findings of NBBt-test is 100% (11/11) and the true finding ratios of DESeq2 and edgeR tests are 81.8% (9/11) and 72.7% (8/11), respectively.

**Table 1:** qPCR validation of 6 genes with differential poly(A) sites found by statistical methods.

Gene	Poly(A) site (bp)	qPCR (ΔΔ*Ct*)	edgeR		DESeq2		NBBt-test		Inferred true DE
			Statistic	FDR < 0.05	Statistic	FDR < 0.05	Statistic	FDR < 0.05	Isoforms^a^
UBL3	30,339,396	0.54*	1.096	No	0.832	No	0.965	NO	No
	30,339,747		1.196	No	1.388	No	4.465	NO	No
	30,338,534		1.638	Yes	2.868	No	13.939	Yes	Yes
MST123	38,270,255	2.42*	3.536	Yes	6.678	Yes	10.363	Yes	Yes
	38,270,363		2.234	Yes	2.784	No	16.892	Yes	Yes
	38,268,658		2.189	Yes	2.493	No	3.794	No	No
CD47	107,765,916	− 0.32*	− 1.391	No	− 4.637	Yes	− 5.874	Yes	Yes
	107,762,147		− 1.150	No	− 3.293	Yes	− 4.757	Yes	Yes
BCL11B	99,635,630	3.84*	− 1.272	No	− 4.629	Yes	− 7.429	Yes	NA
	99,637,753		− 1.364	No	− 3.667	Yes	− 6.669	Yes	NA
KIAA0465	39,952,652	1.57*	1.519	Yes	3.589	Yes	12.246	Yes	Yes
	39,951,895		1.134	No	2.223	No	2.466	No	No
TESK2	45,809,556	0.21	2.412	Yes	3.032	Yes	3.088	No	No

$\Delta \Delta Ct=\Delta CTT-\Delta CTC$ where $\Delta CTT=TT-HT$ and $\Delta CTC=TC-HC$. Here TT and TC are, respectively, qPCR values of tested gene in treatment and control experiments; HT and HC are, respectively, qPCR values of tested gene and housekeeping gene in treatment and control experiments. Statistic of each method was obtained from count data of RNA reads involving poly(A) sites within each gene. qPCR data was provided by Dr. Neilson.

*Difference between stimulated (48 h) and rest status of T-cells is significant under $\alpha$ = 0.05.

^a^If a gene has significant qPCR(ΔΔ*Ct*) under $\alpha$ = 0.05 and two of three methods also find that a RNA isoform within this gene is differentially expressed between stimulated and rest statuses under FDR = 0.05, then this isoform is defined as differentially expressed (DE).

### Identification of differential splicing events

**Figure 3:**
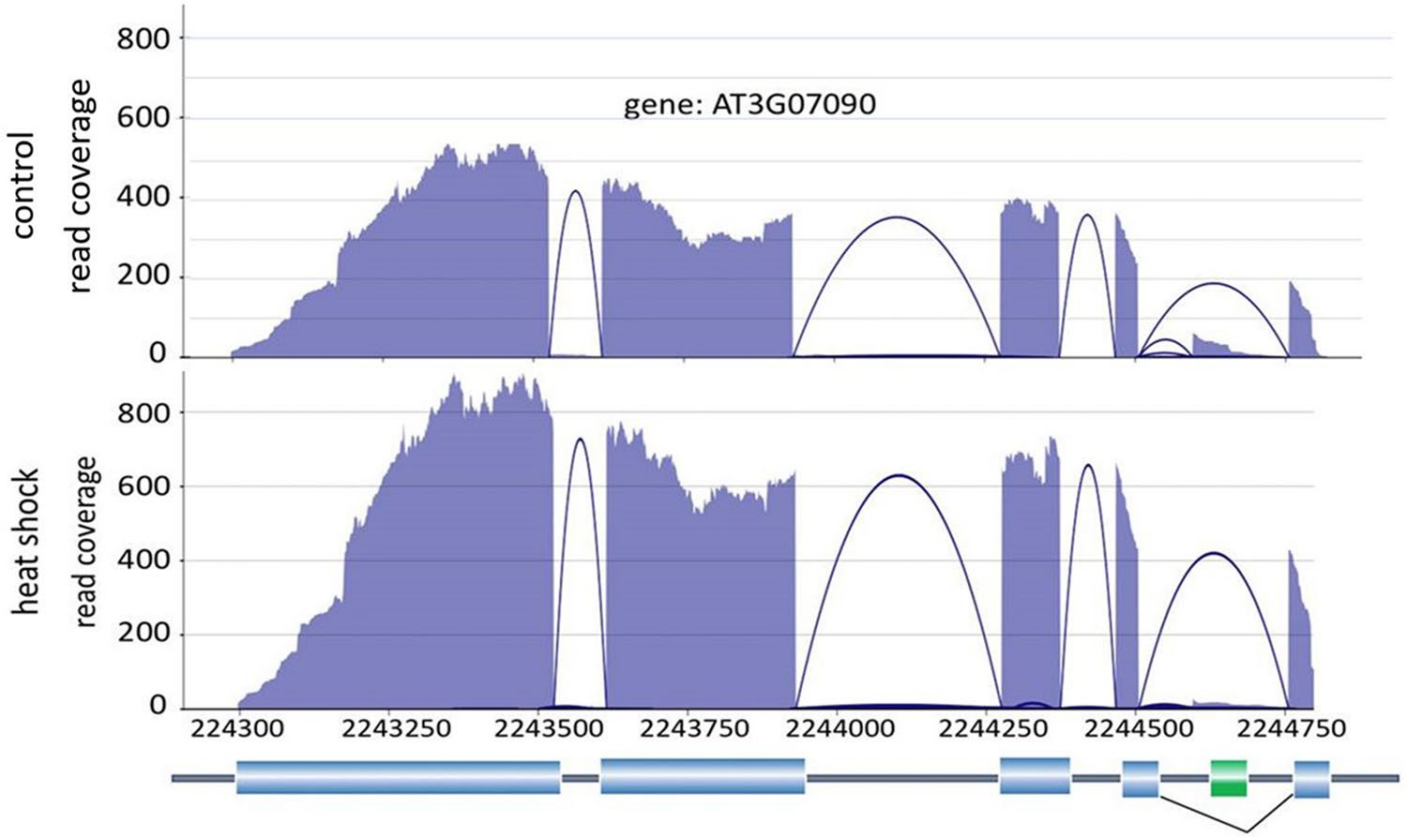
An example of exon splicing event within the gene, AT3G07090 found by spladder-NBBt-test in Arabidopsis RNA-seq data using TAIR10 reference genome. The RNA-seq coverage and splicing pattern for both the control (**a**) and heat shock (**b**) samples are shown along with the annotated transcript model where dark grey lines are introns, blue cylinders are exons and green cylinder is a spliced exon in heat shock samples.

After mapping Arabidopsis RNA-seq data^50^ to TAIR10 genome using STAR aligner^51^, we ran Spladder^11^ on the bam files to produce read coverage data of RNA isoforms. We used NBBt-test to test for differential splicing events at 3′UTR, 5′UTR, skipping exon, intron retention and multiple skipping exons. As an example, NBBt-test found a skipping exon between 2,244,500 and 2,244,750 bp within gene AT3G07090 in the heat shock samples (Fig. 3b) compared to control samples (Fig. 3a), while DEXSeq did not find it.

**Figure 4:**
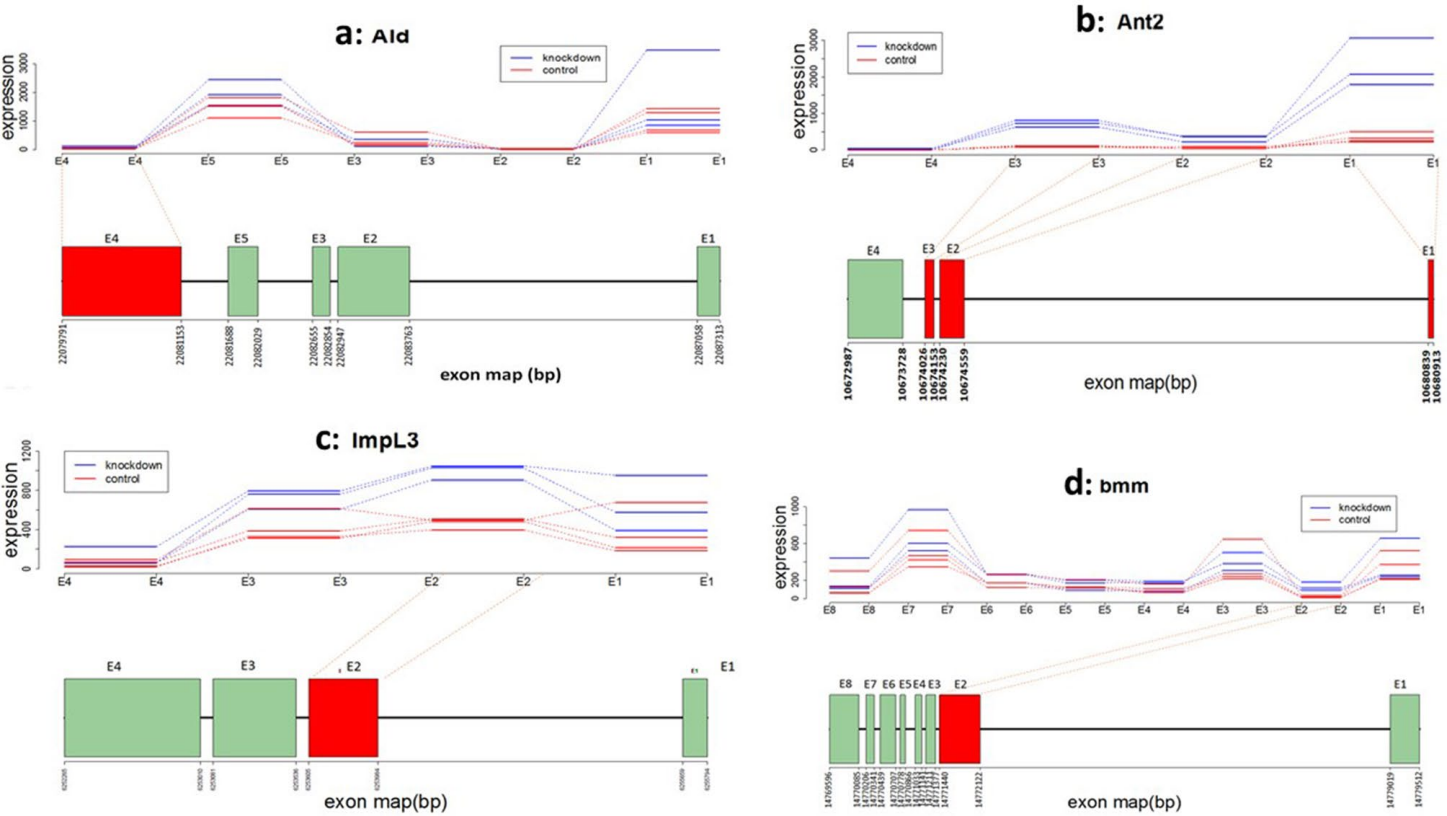
NBBplots for differential expression of exons within genes detected in *Drosophila melanogaster.* Top part in each panel is expression (count in y-axis) of exons within the gene where three red lines stand for three replicates of control (no pasilla knockdown) in the *Drosophila melanogaster* and four blue lines represent four replicates of pasilla knockdown. Bottom part of each panel is a map of exons and introns. Red box shows significant differential expression of the exon between knockdown and control samples found. In gene *Ald* (**a**), DE exon1 (E1) was identified by DEXSeq only. In gene *sesB/Ant2* (**b**), both DEXSeq and NBBt-test identified exons E1, E2 and E3 differently expressed between control and pasilla knockdown. In gene *lmpL3* (**c**), exon E2 was found by NBBt-test only to have differential expression. In gene *bmm* (**d**), exon E2 was found by both DEXSeq and NBBt-test to be differentially expressed. Genes *sesB/Ant2* and *bmm* were validated by RT-PCR^53^. Data from Brooks et al.^53^. Red box represents differential expression exon and green box represents no differential expression exon.

**Figure 5:**
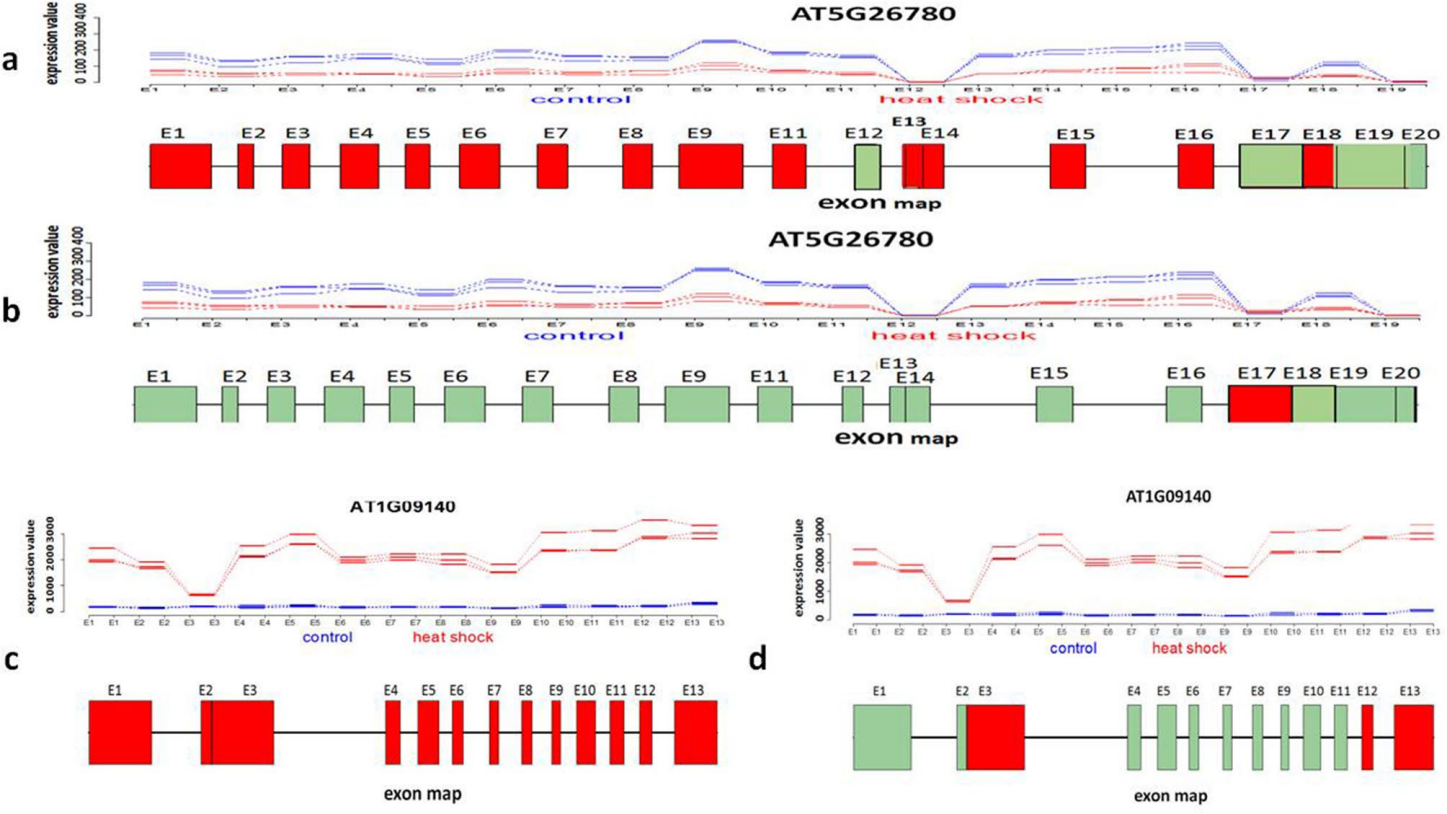
NBBplots for differential expression of exons within genes AT5G26780 and AT1G09140 in *Arabidopsis*. Top part in each panel is expression (count of reads in y-axis) of exons within the gene where three red lines stand for three replicates of control in the *Arabidopsis* and three blue lines represent three replicates of heat shock. Bottom part is a map of exons and introns. Red box shows differential expression of the exon between heatshock and control samples found. (**a**) Differential expressions of exons E1–11, E14–E16, E18 within gene AT5G26780 between heat shock and control samples were identified by NBBt-test. (**b**) Exon E17 within gene AT5G26780 was identified by DEXseq to have differential expression between heat shock and control samples. (**c**) All 13 exons within gene AT1G09140 were identified to be differentially expressed between heat shock and control samples by NBBt-test. (**d**) Differential expression of exons E3, E12-E13 within gene AT1G09140 between heat shock and control samples were identified by DEXSeq. Genes AT5G26780 and AT1G09140 were validated to have exons differential expression by experiment.

**Figure 6:**
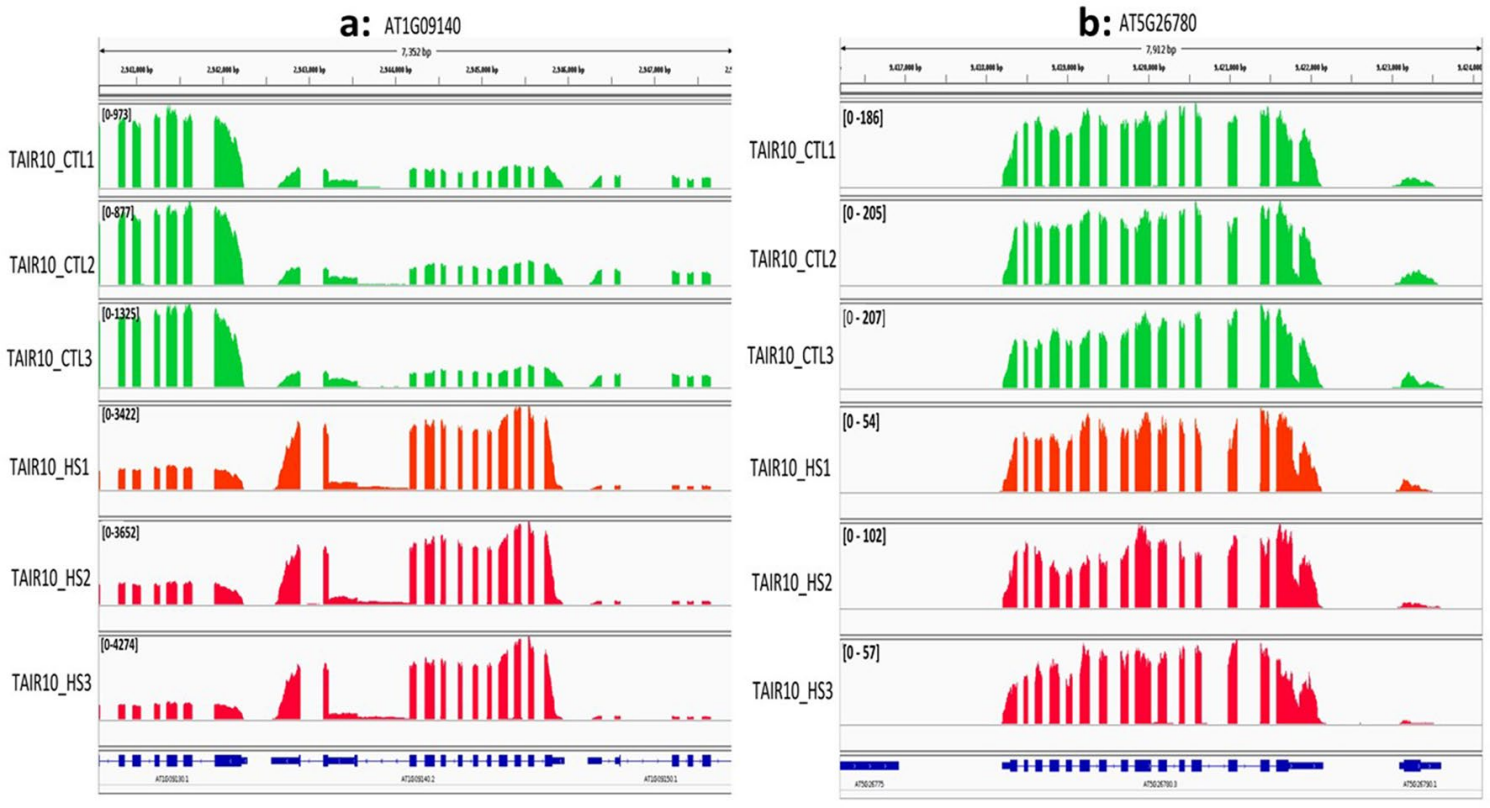
Mapping plots of *Arabidopsis thaliana* gene AT5G26780 and AT1G09140. Left panel is IGV plots of *Arabidopsis thaliana* gene AT5G26780 (**a**) where 16 exons in gene AT5G26780 show differential mapping of reads between control replicates and heat shock replicates. No exons E12 and E19 were found. Exon E17 is actually an intron between Exons E15 and E16. Control replicates have read counts in region of 180–207 while Heat shock replicates show 54–80 read counts. Right panel is IGV plots of *Arabidopsis thaliana* gene AT1G09140 where 13 exons in gene AT1G09140 (**b**) show differential mapping of reads between control replicates and heat shock replicates. Exons E2 and E3 were connected together. Control replicates have read counts in region of 180–207 while heat shock replicates show 54–80 read counts.

We performed DEXSeq^31^ and NBBt-test on exon count data obtained by applying HTseq^52^ to map RNA-seq reads^53^ to *D.melanogaster* genome. As examples, we here just displayed exon expression profiles of genes *Ald, Ant2, lmpL3*, and *bmm* using NBBplot: pasilla counts of knockdown and control replicates in DE exon E1 (red box) within gene *Ald* identified by DEXSeq only are not separated (Fig. 4a). However, in gene *lmpL3* (Fig. 4c), DE exon E2 (red box) found by NBBt-test only shows clear separation replicate counts between control and pasilla groups. DE exons E1, E2 and E3 (red boxes) in gene Ant2 (Fig. 4b) and exon E2 (red box) within gene *bmm* (Fig. 4d) identified by both DEXSeq and NBBt-test show significantly more counts of reads in the three pasilla knockdown replicates than in the four control replicates. The differential expression of exons E1, E2, and E3 within gene *Ant2* and of E2 within gene bmm were validated by qPCR^53^. Similarly, Fig. 5 displays big differences between NBBt-test and DEXSeq in the identification of differentially expressed exons within Arabidopsis genes, AT5G26780 and AT1G0914055. Gene AT5G26780 has 20 exons, except for exons E12, E17, E19, and E20, NBBt-test found that the other 16 exons showed differential expression between control and heat shock groups (Fig. 5a), while in DEXSeq findings, only exon E17 had differential expression (Fig. 5b). However, the observed data show that exon E17 had no difference between the control and heat shock groups. Figure 6b shows that gene AT5G26780 had higher read coverage in the control group (186, 205, 207) than in the heat shock group (54, 102, 57), which are well consistent with read counts of exons shown in Fig. 5a or b. In gene AT1G09140 (Fig. 6a), NBBt-test found that all 13 exons had differential expression (Fig. 5c) and the read coverage data (Fig. 6a) show this result that three heat shock(HS) replicates had many more read counts (3422, 3652, 4274) than three replicates (973, 877, 1325) of control group (CTL) but DEXSeq was able to find only three DE exons (E3, E12 and E13) (Fig. 5d) while NBBt-test found them all (Fig. 5c). Differential splicing events of these two Arabidopsis genes were previously validated by qPCR experiment^54^.

### Differential CRISPR screen analysis

**Figure 7:**
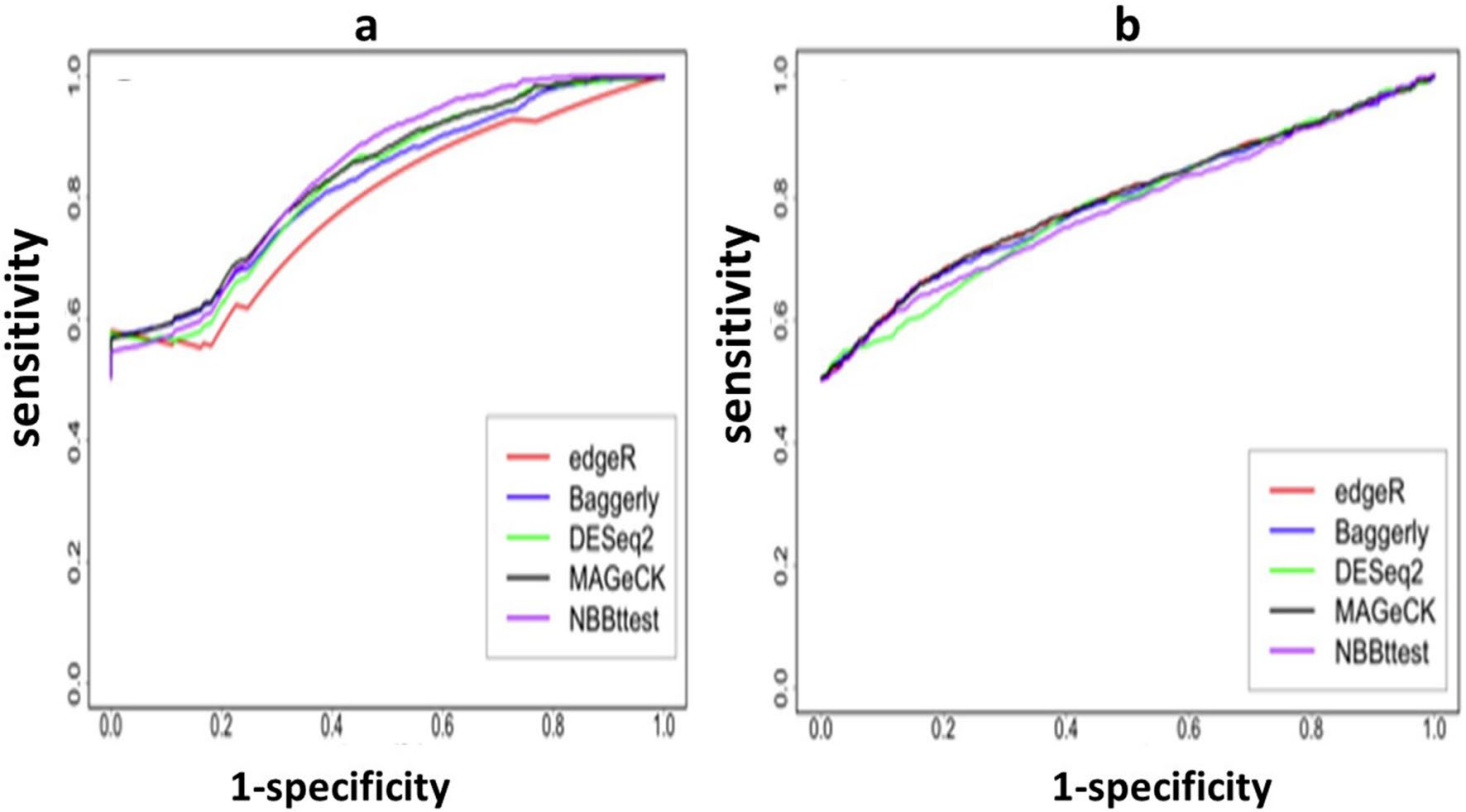
Performances of statistical methods in identifying differential knockout screening sgRNAs targeting genes. ROC curves display performances of the statistical methods edgeR, DESeq2, beta t-test of Baggerly et al. MAGeCK and NBBt-test for identifying truly differentially screening sgRNAs targeting genes between T1 and T0 in the CRISPR screen spike-in data from cell lines RT112 (**a**) and UMUC3 (**b**), respectively.

**Figure 8:**
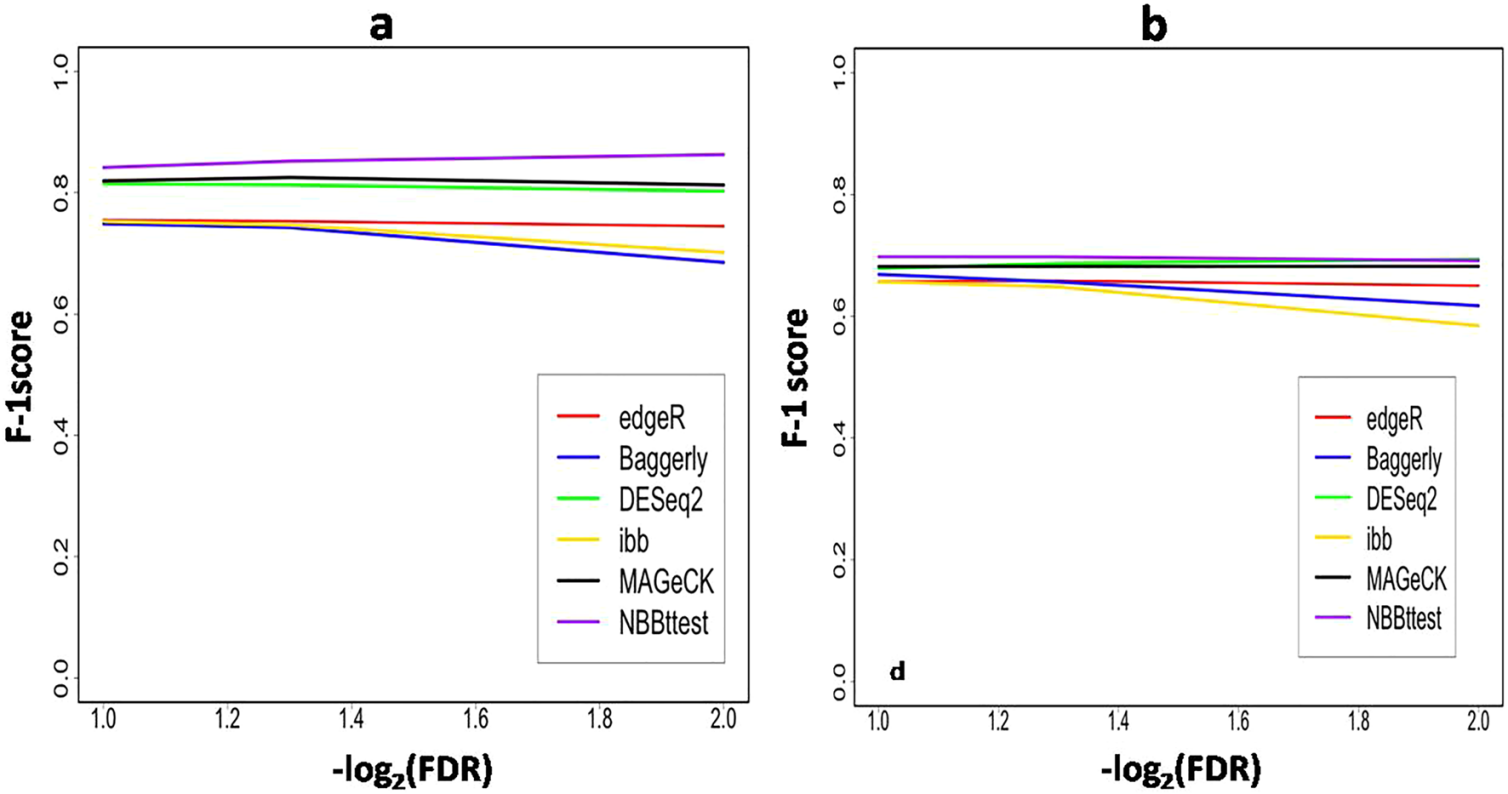
F1-scores of statistical methods in identifying differential sgRNAs. F1-score is defined as the ratio between recall $\times$ precision to recall + precision (see Methods), or the harmonic mean of precision and recall. Panel a shows F1-scores of the selected methods edgeR, DESeq2, beta t-test of Baggerly et al. ibb, MAGeCK and NBBt-test in finding differential hits of sgRNAs targeting DNA sequences of genes detected in cell line RT112 across − log10 = 1–2 and Panel b shows F1-scores of these six statistical methods in identifying differential hits of sgRNAs targeting DNA sequences of genes detected in cell line UMUC3.

**Figure 9:**
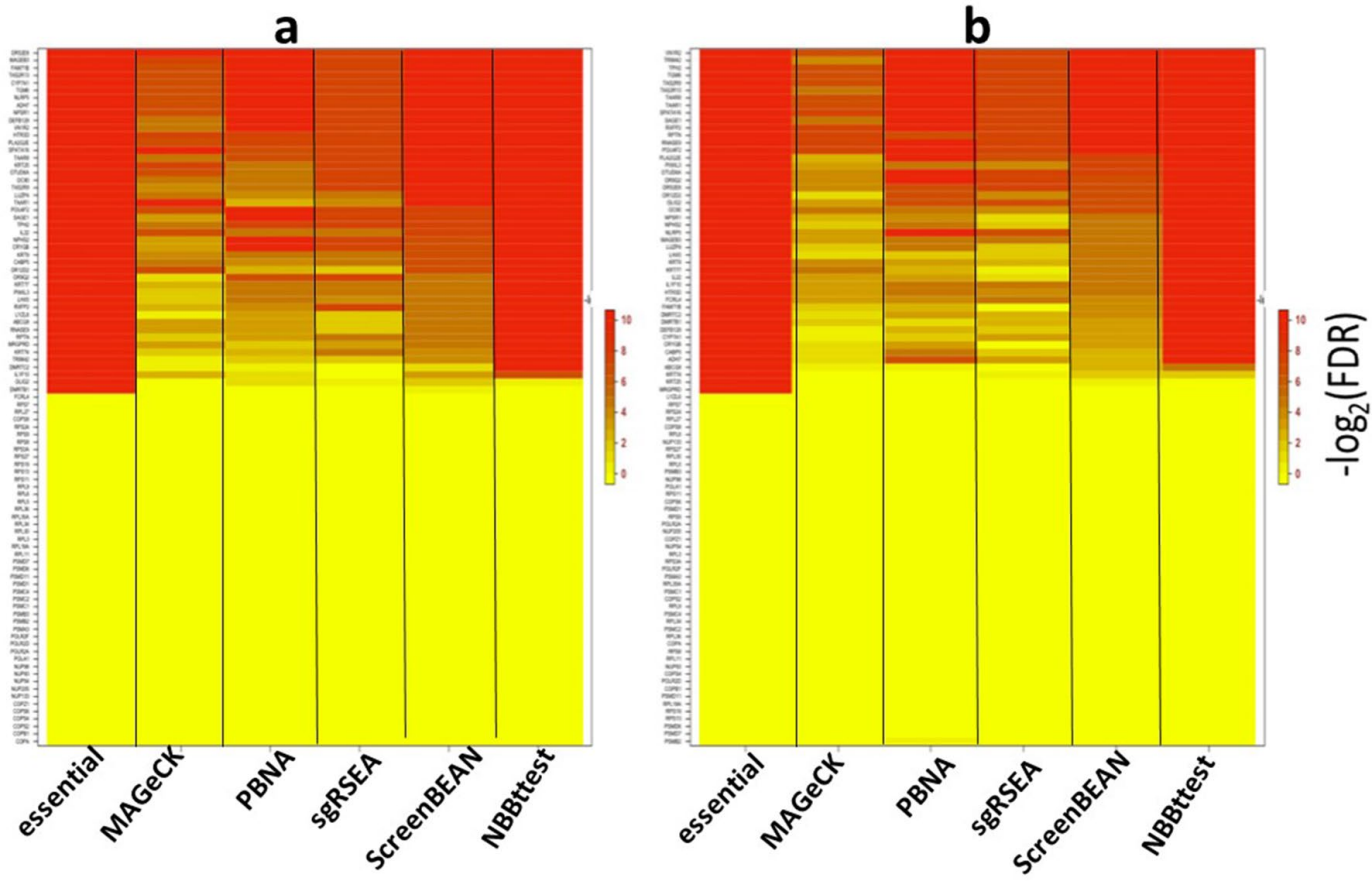
Heatmaps for the observed − log_2_(FDR) of the 6 statistical methods in identifying differential screening genes targeted by sgRNAs. Heatmaps for the observed − log_2_(FDR) at which differential screening genes targeted by sgRNAs in cell lines RT112 (panel **a**) and UMUC3 (panel **b**) were identified by a statistical method. Log_2_(FDR) = 0 means FDR = 1, − log2(FDR) = 2.995732 means FDR = 0.05, − log_2_(FDR) = 6.643856 means FDR = 0.01, and − log2(FDR) = 9.965784 means FDR = 0.001. To compare these 6 methods in identifying differential screen genes targeted by sgRNAs, we set − log_2_(FDR) = 9.965784 if a gene is essential, − log_2_(FDR) = 0, otherwise. Data obtained from Evers et al. (2015).

For the differential CRISPR knockout screens, we used datasets from Evers et al^55^ that have two spike-in datasets RT112 and UMUC3 where there are 93 genes each targeted by 10 sgRNAs and 48 of 93 genes were known to be essential genes. Along with NBBt-test, the other four statistical methods were chosen to perform differential analysis of CRISPR knockout screens. These methods are edgeR^23,24^, Baggerly et al.’s beta t-test (called Baggerly)^41^, DESeq2^22^, NBBt-test and MAGeCK^18^. DESeq2, edgeR and Baggerly are general methods for differential analysis of RNA-seq data while MAGeCK was specifically developed for testing differential CRISPR knockout screens at either gene or sgRNA levels. For RT112 data, the essential genes are clearly separated from non-essential genes (Supplementary Fig. S1a), receiver operating characteristic (ROC) curves showed that NBBt-test had the best performance among these five methods, while the edgeR had the poorest performance (Fig. 7a). Figure 8a shows that NBBt-test had the highest F1-score (see Methods section), followed by MAGeCK, suggesting that NBBt-test had the highest precision and the highest recall among these methods. Baggerly and ibb methods reported the lowest scores. However, for UMUC3 data, there is no significant difference in the performance among these methods (Fig. 7b) but NBBt-test still had the highest F1-score across − log10(FDR) = 1 to 2 even though the F1-scores are less than 0.7. MAGeCK and DESeqa2 had the same F1-score (Fig. 8b). This is probably due to low quality of the UMUC3 data where 135 of 486 essential sgRNA targets are false and mixed up with the non-essential sgRNA targets (Supplementary Fig. S1b). This is also seen at gene level (Supplementary Fig. S2): For RT112, all these five methods had a perfect performance (Supplementary Fig. S2a) but in UMUC3 data, except that MAGeCK had a perfect performance, NBBt-test had the best performance among the other methods (Supplementary Fig. S2b). We created heatmaps (Fig. 9a,b) to display − log_2_FDR profiles of these five statistical methods where in the essential column, gene had FDR = 0 (red) if it is essential, otherwise, FDR = 1(yellow). The heatmaps show that NBBt-test is the best method to find essential genes based on RT112 (Fig. 9a) and UMUC3 (Fig. 9b) data.

### Simulation comparison

**Figure 10:**
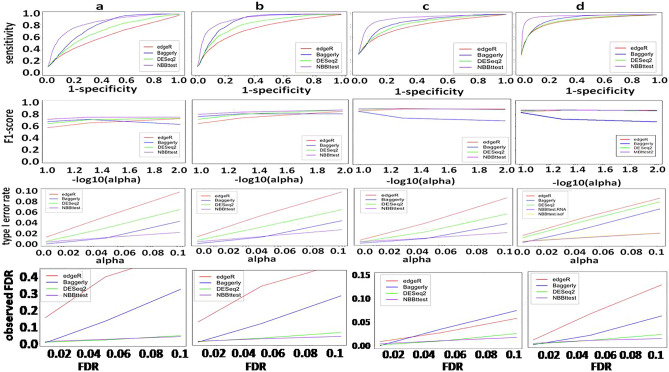
Performance, F1-score, and type I error rate of statistical methods in finding differential polyadenylation sites. ROC curve, F1-score and type I error rate and observed FDR of a method were given by performing this method on the simulated poly(A) count data. F1-score and type I error rate were calculated based on single-test results. The observed FDR (FDR(o)) under significant cutoff is used to compare theoretical FDR(FDR(t)). In an ideal case, FDR(o) = FDR(t), the line is a diagonal line, suggesting that FDR is correctly estimated. So, if the line is over the diagonal line, then, the FDR is underestimated, more false discoveries were not estimated. If the line is below diagonal line, then the FDR is overestimated, that is, estimated false discoveries are more than true false positives. The simulated poly(A) count data were generated by using negative binomial distribution on the Jurkat T-cell poly(A) count data. We simulated four scenarios A, B, C and D. Each scenario had two samples with equal sample size of 3 and 13. 409 poly(A) sites were scattered in 9294 genes and **30%** artificial noise (or outliers). (**a**) **10%** of poly(A) sites were positively or negatively responded to **100*U*** stimulation effect. (**b**) **10%** of poly(A) sites were positively or negatively responded to **300*U*** stimulation effect. (**c**) **30%** of poly(A) sites were positively or negatively responded to **100*U*** stimulation effect. (**d**) **30%** of poly(A) sites were positively or negatively responded to **300*U*** stimulation effect where 0 < $U\le 1$.

To fully evaluate our NBBt-test, we designed multiple scenarios to simulate poly(A) count data, splicing junction count data and FACS data of CRISPR screens as described in the Methods section. For the count data of poly(A) RNA reads, we designed four scenarios to simulate stimulated and normal statuses each having 3 replicates with 30% technical noise (or outliers). We used the simulated datasets in these four scenarios to compare NBBt-test to DESeq2, edgeR, and the Baggerly methods. The results show that NBBt-test had the best performance (ROC curves), the highest F1-score, the lowest type I error rate and the lowest observed FDR among these four methods (Fig. 10). We also simulated data of two samples each having 5 replicates with 30% technical noise of 13,409 poly(A) sites scattered in 9,294 genes and each having 3 replicates without technical noise in four scenarios (**a: 10%** of poly(A) sites were positively or negatively responded to 100U T-cell stimulation effect; **b:** 10% of poly(A) sites were responded to 300U stimulation effect; **c:** 30% of poly(A) sites were responded to **100U** stimulation effect. **d:** 30% of poly(A) sites were responded to 300U stimulation effect where U is a uniform variable, 0 < U $\le 1$). We used the first set of simulated datasets to compare NBBt-test, DESeq2, edgeR and Baggerly. The results are summarized in Supplementary Fig. S3. In these four scenarios with technical noise, NBBt-test had the highest performance among these five methods (Supplementary Fig. S3), the highest F1-scores, the lowest type I error rates and the lowest observed FDRs in all these four scenarios (Supplementary Fig. S3). For the second set of simulated data, both NBBt-test.RNA (test for differential expression of RNA species or gene expressions), NBBt-test.isform (test for differential expression of RNA isoforms) also had the highest performance (ROC), the highest F1-score, the lowest type I error rate and the lowest observed FDR in all four scenarios (Supplementary Fig. S4) among these methods.

We also simulated FACS data of CRISPR knockout screen to compare NBBt-test, edgeR, DESeq2, Baggerly, and MAGeCK in 8 scenarios (see Methods section). Likewise, NBBt-test still had much better performance than the other four methods in all these 8 scenarios (Supplementary Fig. S5). Except for scenarios g and h, NBBt-test had the highest F1-score across − log10($\alpha$) in the other 6 scenarios (Supplementary Fig. S6) and had type I error rate of 0 from $\alpha$ = 0.0 to $\alpha$ = 0.1 except that NBBt-test had type I error < 0.005 in scenarios a and b (Supplementary Fig. S7). MAGeCK, edgeR and DESeq2 also showed type I error rate = 0.0.

In another experiment, we simulated count data of splicing events in two samples using negative binomial generator in R environment based on pasilla exon splicing count data where there are 69,733 exons distributed within 14,206 *Drosophila* genes. Control sample has 4 biological replicates and knockdown sample has 3 biological replicates. In the all four scenarios, NBBt-test outperformed DEXSeq and had F1-score = 100% and the observed FDR = 0 from FDR = 0 to 0.1 (Supplementary Fig. S8), while DEXSeq had F1-score = 44% and the observed FDR = 0.65 from FDR = 0 to 0.1(Supplementary Fig. S8).

## Discussion

NBBt-test is designed to identify differential isoforms or CRISPR screen sgRNAs by taking advantage of three important features of RNA-seq data. In the first case, we assume that RNA sequencing is a negative binomial event with a probability (*p*) that follows beta distribution with parameters, α and β, which is generally accepted in RNA sequencing^23,24^. We employed a modified algorithm of Baggerly et al.^41^ to estimate parameters, α, β and *p*. The second feature is that RNA-Seq experiments are mostly conducted in very small samples (mostly less than 8 biological replicates), while small samples easily result in data gaps between two different conditions. In theory, two count datasets that are separated with a gap have higher probability that they come from two different distributions than those that overlap. For this reason, we introduced a gene-specific or isoform-specific variable $\rho$ into the beta t-test to control false discoveries. $\rho$ is used to measure the overlap between two count datasets and data homogeneity. If two count datasets overlap more and/or have bigger within-condition variances, then $\rho$ < 1, otherwise, $\rho$ > 1 for separating and homogeneous data. Thus, $\rho$ shrinks *t*-values for overlapped count datasets and inflates *t*-values for clearly separated count datasets with small noises. To consider sample size effect, we set a threshold $\omega_{\alpha }$ for $\rho$. That is, *t*-statistic is inflated with $\rho$ > *ω* or shrunken with $\rho$ < $\omega_{\alpha }$. As the result, most of the *t*-values with $\rho$ < $\omega_{\alpha }$ are compressed into small values while those with $\rho$ > $\omega_{\alpha }$ are enlarged. Since *p*-value only depends on *t*-value given the degree of freedom, *p*-values with inflated *t*-values become smaller while those with shrunken *t*-values become larger, so very few false positives would be found. Threshold $\omega_{\alpha }$ is an estimate of null $\rho$ value. $\omega_{\alpha }$ is a dependent of sample size and data quality. Our simulation shows that the smaller the sample size is, the larger the $\omega_{\alpha }$ is than $\rho$ (Fig. 1d). However, when the sample size reaches 15 replicates, $\omega_{\alpha }$ is only slightly greater than $\rho$. These results are expected because in the case of sample size < 15, the probability that data gap occurs between conditions is inversely proportional to sample size (Fig. 1c), then $\rho$ is smaller than $\omega_{\alpha }$. $\rho$ < $\omega_{\alpha }$ leads to a result that the new t-statistics < the old t-statistics, implicating that t-statistics is shrunken and *p*-value increases. In the case of sample size ≥ 15, however, the data gap is vanished, $\rho$ $\cong$  $\omega_{\alpha }$, the new t-test is reduced to the old one. For the third feature, we used a gene-level statistic to furthermore correct bias of *t*-statistics. We used the sum count over all isoforms as count of a gene. We created a new *t*-statistic to test for differential expression of genes or isoforms. The new t-test includes three parts: classic t-test, fold change (FC), and F-like test. In Eq. (2), $\varphi_{gi}$ is a strict fold change or nonparametric U-test for two overlapping data. Both are two components of $\rho$ and in Eq. (3), $\zeta_{gi}$ is similar to F-statistic. $\varphi_{gi}$ controls false discoveries or false positives by measuring data gaps between conditions while $\zeta_{gi}$ controls noise or within-group variations. These are the reasons why NBBt-test outperforms the other differential methods. We can predict that even though $\rho$ loses false discovery control in large samples (sample size *m* > 15), *t*-test would still have higher statistical power than the other compared methods. Therefore, *t*-test can find differential isoforms/sgRNAs or genes in either small samples or large samples with low type I error and high power.

## Methods

### RNA-seq data collection

#### Poly(A) RNA-seq data

Polyadenylated (Poly(A)) RNA sequence data were derived from Jurkat T-cells of mouse stimulated with plate-bound antibodies. Total RNA was harvested from resting and stimulated cells with Trizol reagent as per manufacturer instructions. Poly(A) RNA was isolated with the Poly(A)-Purist MAG kit as per manufacturer instructions. High-throughput sequencing libraries were generated essentially as described in reference^2^, with the exception that “barcoded” linkers were used to facilitate multiplexing. Libraries were sequenced via 50 bp paired-end sequencing on an Illumina GAIIx. The data were derived from Tan et al^42^.

#### CRISPR knockout screen count data

Evers et al.^55^ compared performances of CRISPR knockout screening, shRNA, and CRISPRi in discriminating hits from non-hits in functional genetic screens and published their CRISPR knockout screen data on RT-112 and UMUC-3 cell lines. This study chose 93 genes that consistently show low expression and do not affect phenotype upon knockdown for knockout screens. Of these 93 genes, 46 selected from COP9 signalosome (7 genes), proteasome (10 genes), nuclear pore complex (5 genes), ribosomal complex (20 genes) and RNA polymerase complex (4 genes) were considered essential for both cell lines. From the non-essential genes identified by Hart et al.^13,28^. Genes that are expected to have very consistent phenotype are defined as non-essential^55^. Each gene was hit by a set of 10 sgRNAs. These two CRISPR knockout screening count datasets were downloaded from Evers et al.^55^.

#### Heat shock RNA-seq data

RNA-seq data derived from *Arabidopsis thaliana* plants subjected^50^ to heat shock treatment at time points T1 (heat shock period) and T2 (recover period) and control were downloaded from SR45a RNA-binding (RRM/RBD/RNP motifs) family protein [Arabidopsis thaliana (thale cress)] - Gene - NCBI (nih.gov)  and also from https://www.arabidopsis.org/servlets/TairObject?type=gene&name=AT1G07350.1.

#### Pasilla RNA-seq count data

The mammalian proteins, NOVA1 and NOVA2 (collectively named here as NOVA1/2) are perhaps the best-characterized splicing regulators to date. NOVA1/2 encodes RNA binding proteins with three KH-domains that recognize clusters of YCAY repeats^53^. The gene, pasilla (PS), the *Drosophila melanogaster* ortholog of mammalian NOVA1 and NOVA2, is well-studied for its splicing regulation. To study the impact of splicing regulators on splicing events, the gene pasilla, a splicing regulator in *D. Melanogaster,* was knocked down with RNAi. To explore PS-regulated exons, RNA-seq was used to identify splicing events that changed upon depletion of PS knocked down with RNAi. Libraries were prepared from RNA extracted from seven biologically independent *D. melanogaster* cell samples: three control samples and four PS knockdown samples and deep sequenced on an Illumina Genome Analyzer II (GAII), partly using single-end and partly paired-end patterns at various read lengths^53^. The RNA-seq sequence reads are available for download from the NCBI Gene Expression Omnibus (http://www.ncbi.nlm.nih.gov/geo) using accession numbers GSM461176–GSM461181. RNA-seq count data were downloaded from https://genome.cshlp.org/content/21/2/193/suppl/DC1.

#### qPCR data for validating differential poly(A) isoforms

qPCR data for validating differential poly(A) isoforms^42^ were obtained by isolating total RNA from resting and stimulated (48 h) Jurkat T-cells and performing real-time PCR in triplicates with gene-specific primers and the Bio-Rad SYBR FAST iCycler qPCR kit (Kapa Biosystems) on a Bio-Rad CFX96 real-time thermal cycler. Here 6 genes selected are UBL3, MST123, CD47, BCL11B, KIAA0465 and TESK2, among which genes UBL3 and MST123 have 3 poly(A) sites, CD47, BCL11B, and KIA0465 have two poly(A) sites and TESK2 without multiple poly(A) sites. The ΔΔC_T_ method was used to calculate expression relative to TBP (ATA-box binding Protein). The qPCR data were derived from Tan et al^42^.

### Simulation study

#### Simulation of poly(A) RNA count data

Since RNA sequencing was assumed to follow negative binomial distribution (NB), we used the NB pseudorandom generator to create poly(A) RNA isoform count datasets. Simulations were performed in R-environment by using NB($\mu$ + $\tau$,s) and NB($\mu$, s) where $\mu$ and s are respectively mean and dispersion parameter (here is variance) for per poly(A) RNA isoform solved from experimental poly(A) RNA isoform count data from Jurkat T-cell in two conditions (resting and stimulating statues) each having 3 replicated libraries, where 18,290 isoforms scattered in 9572 genes were collected. We set two levels (A = 100 and 300) of treatment effect impacting differential expression of poly(A) isoforms and linearly and randomly assigned effect size $\tau = UA$ to 10% and 30% null poly(A) isoforms where U is uniform variable ($U \in (0,1]$), so $\tau$ is linearly distributed from > 0 to A. In addition, 30% of the null poly(A) isoforms were assigned with outliers (technical). Equal sample sizes were set as 3 and 5 replicate libraries.

#### Simulation of exon count data

Similarly, we still assumed that exon RNA sequencing follows negative binomial distribution. In R-environment, we used NB pseudorandom generator to create exon RNA isoform count datasets. The simulation was conducted by using parameters per exon RNA isoform solved from experimental exon RNA isoform count data from *D. Melanogaster* in two conditions (control and PS knockdown) where control has 4 replicate libraries and PS knockdown has 3 replicate libraries and 69,733 exons were scattered in 16,206 annotated *Drosophila* genes. We set two levels (A = 100 and 300) of PS knockdown effect on differential splicing events and linearly and randomly assigned the effect size $\tau = UA$ to null exon isoforms with 10 and 30% probabilities, where U is uniform variable ($U \in (0,1]$), switch ratio = 0. Thus, we simulated 4 scenarios: Scenario **a**: knockdown effect size A = 100U, 10% of exons were differentially spliced; Scenario **b**: knockdown effect size A = 100U, 30% of exons were differentially spliced; Scenario **c**: Knockdown effect size A = 300U, 10% of exons were differentially spliced; Scenario **d**: Knockdown effect size A = 300U, 30% of exons were differentially spliced.

#### Simulation of data of CRISPR FACS screen

Genetic screening is a powerful discovery tool that provides an important functional complement to observational genomics. Several platforms for mammalian cell screens have been developed based on CRISPR technology^18,56^. For example, CRISPR nuclease (CRISPRn) screens^15,19,20,57^ interfere gene function by targeting Cas9 nuclease programmed by a sgRNA to the coding region of a gene of interest in a genome, and followed by error-prone repair through the cellular non-homologous end-joining pathway, while CRISPR interference (CRISPRi) and CRISPR activation (CRISPRa) screens^58^ repress or activate gene transcription by exploiting a catalytically dead Cas9 to recruit transcriptional repressors or activators to their transcription start sites, as directed by sgRNAs. There are two distinct strategies for phenotypic selection: Fluorescence activated cells sorting (FACS)-based screens in which cell populations are separated based on a fluorescent reporter signal that is a function of the phenotype. Another strategy is growth screens in which the pooled screens are conducted to select genes with growth phenotype by comparing cell populations at an early time point with cells grown in the absence or presence of selective pressures, such as drugs or toxins. To evaluate these differential methods for identifying differential hits of sgRNAs, we here simulated data of CRISPR FACS screens using CRISPulator tool^59^ in 8 scenarios consisting of combinations of three parameters each with two levels: FACS bins (0.1, 0.25), noises (0.5, 1.0), phenotype proportions (0.2, 0.4). The FACS bin is percent of cells in “low” and “high” population. Noise indicates deviation of Gaussian distribution, for example, noise = 0.5 means $\sigma$ = 0.5, a normal distribution with deviation of 0.5. Phenotype proportion is fraction of genes with negative and positive phenotypes; for example, phenotype proportion = 0.2 means that 20% of genes show negative and positive phenotype and 80% of genes show null (or neutral) phenotype. In each scenario, we set number of genes = 1000, coverage (number of sgRNAs per gene) = 10 and two conditions each having equal sample size of 4. After simulation, we implemented MAGeCK^18^ to map the RNA sequence reads to a reference genome, annotate genes with phenotypes, and create hit count data.

### Computational programs and pipeline analysis of RNA-seq data

**Figure 11:**
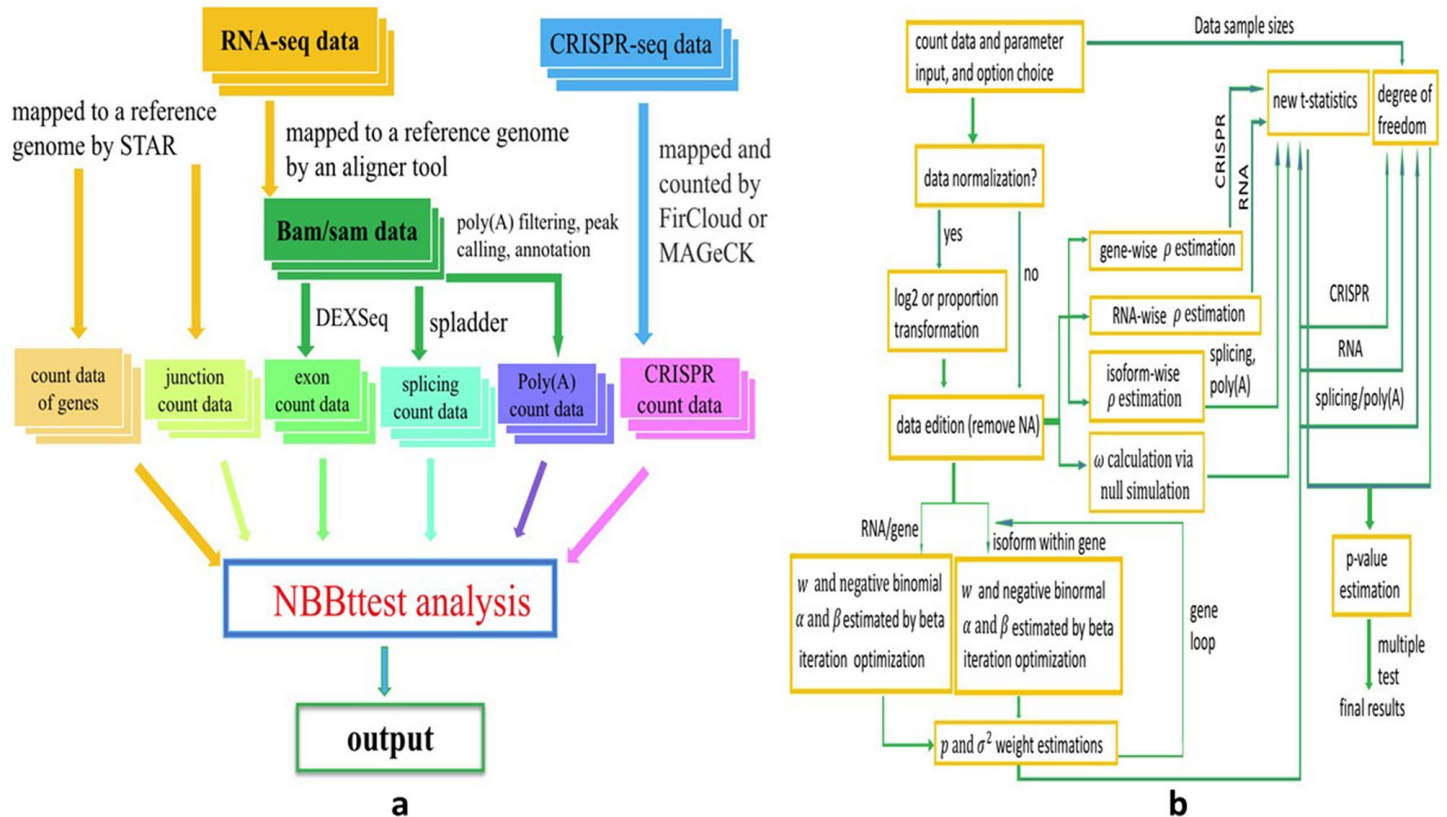
Computational procedures and workflow of NBBt-test. (**a**) Workflow of NBBt-test. RNA-seq data have two types: RNA-seq reads and CRISPR-seq reads. RNA-seq reads are mapped to a reference genome such as mm9 or mm10 in mice, or hg19(GRCh37) or GRCh38 in human. Currently, popular aligners are STAR, Tophat, BWA, and Boetie. STAR can use RNA-seq data such as fastq and fasta to map RAN-seq reads to reference genome with STAR index. STAR outputs count data of genes, and sorted bam data, For studying splicing, one needs another tool such as spladder, rMATS, cufflinks, to remap bam file to annotated genome file such as hg.gtf or hg.gff3. CRISPR-seq reads are mapped to genomes by MAGeCK to generate count data. (**b**) Computational procedures. In this workflow, first, the count data (RPKM, FPKM or TPM) is normalized with log2, or proportion transformation (library size is the same across all individuals). Normalized count data are used to estimate *ρ* and *ω* and *α* and *β*, variance V, and proportion *p*. Finally, new t-statistics and *p*-values are calculated to perform multiple tests or adjust *p*-values.

Figure 11a summarized workflow of NBBt-test. As a differential analysis tool, NBBt-test can perform differential analysis of poly(A) RNA-seq, CRISPR knockout screen RNA-seq, splicing RNA-seq, differential exons, RNA-seq(shRNA-seq, mRNA-seq). Figure 11b displays an overview of the computational procedure of the NBBt-test.

### Analysis pipeline for poly(A) RNA-seq data

The paired end RNA reads were mapped to the mouse genome (mm9) using the paired-end mapping module of bwa^60^ with default alignment parameters. Non-mapping reads were remapped to the UCSC KnownGene reference and then projected back to mm9. Individual reads were condensed to tags based on their 3′ coordinate by sliding 20-nucleotide window using the frequency-weighted median 3′ coordinate as the tag identifier. Tags were then filtered using a progressive filtering strategy assessing adenosine and guanine composition in the five, ten, and fifteen bases followed the tag-mapping site and then assigned to individual transcription units. For each transcription unit, the aggregate tags mapping to the unit were ranked based on frequency. Tags were extracted from the highest frequency to the lowest until the extracted tags excessed 90% of the aggregate frequency for the gene or isoform. The remaining tags were discarded^42^. All reads derived from an annotated transcription unit were considered as an individual entity. After that, counts of reads were obtained for an RNA isoform. This pipeline analysis was implemented by polyapipeline (Github).

### Analysis pipeline for CRISPR knockout screen RNA-seq data

Data analysis pipeline for CRISPR screen RNA-seq data was conducted by performing MAGeCK^18^ following the third demo: going through a public CRISPR/Cas9 screening dataset as described in the tutorial (https://sourceforge.net/p/mageck/wiki/demo/#the-second-demo-starting-from-raw-fastq-files). Briefly, MAGeCK requires a library file that lists three columns: sgRNA ID, sequence, and gene without comma in a txt file. So before running MAGeCK, user should make a library.txt file following provided library format. In the current version, MAGeCK can automatically determine the trimming length and sgRNA length, so user does not need to consider trimming RNA sequences. In this pipeline, we used two commands, *count* and *test*. The *count* command produces a count table from MAGeCK containing two columns for sgRNA and gene and the rest of the columns for count data. The *test* command is used to compare two conditions to test for differential genes targeted by sgRNAs or differential sgRNA-targeted genes.

### Analysis pipeline for splicing RNA-seq data

Similar to the general RNA sequence analysis pipeline, for splicing RNA-seq data we performed FastQC^61^ to check for sequencing data quality, trimmed adapters and contaminations, filtered or removed overrepresented sequences or low-quality sequences, and corrected for errors to obtain clean sequencing data using the Galaxy server^62^. We used three pipelines to map splicing RNA-seq reads to the reference genome as described below.

### Star-spladder for splicing events

Firstly, STAR^51^ was used to map RNA reads from samples onto the reference genome using gff3 annotation file and generate sorted bam files. In python 2.7 environment, spladder^11^ was carried out on these sorted bam files with C = 3 (confidence = 3) and gff3 annotation file and ran build model to align and annotate 3′UTR splicing events, 5′UTR splicing events, (cassette) exon skipping events, intron retention, multiple exons skipping and mutual exclusive exons into splicing graph representation. Spladder outputs merge_graphs_confirmed.gff3, .icgc.txt.gz, .pickle, counts.hdf5 files of each splicing type. More detailed instructions for running Spladder and setting parameters can be found at https://spladder.readthedocs.io/en/latest/general.html and https://spladder.readthedocs.io/en/latest/spladder_modes.html.

### STAR-DEXSeq for differential exons

Like **STAR**-spladder, at step 1, **STAR** was performed to map RNA reads onto the reference genome using gff3 annotation file to generate sorted sam files. At step 2, in python 2.7 environment, dexseq_prepare_annotation.py was run on annotation.gff3 to DEXSeq annotation file (e.g., DEXseq.gff). The next step was to run dexseq_count.py on sam files and DEXseq.gff to output count data files with genes and exons annotated. This step was done by using THseq^52^.

### RNA-seq data analysis for differential gene expression

After running star to build start index on a reference genome for mapping RNA-seq data, we started with STAR to run alignReads runModel with setting genes.gtf in hg19, bedGraph, and GeneCounts. STAR outputted gene count data file.

### Benchmarking

For poly(A) RNA-seq count data and general RNA-seq count data, we compared the performance of NBBt-test against those of DESeq2, edgeR, Baggerly’s beta t-test^41^ (called Baggerly for convenience) by implementing R packages, DESeq2 and edgeR installed from Bioconductor. This is because DESeq2 derived from DESeq^26,41^ and edgeR are two popular methods for differential analysis that are also available on the Galaxy server^62^. Baggerly et al.’s beta t-test method is a basis of our NBBt-test, so we chose it as a control for our NBBt-test. The Baggerly method was implemented by using R functions that we wrote based on Baggerly et al^41^. The R functions for implementing Baggerly’s beta t-test were determined by realizing a practical example provided by Baggerly et al^41^. For CRISPR screening data of sgRNA, we chose DESeq2, edgeR, Baggerly, and MAGeCK for comparison against our NBBt-test. MAGeCK was specially designed for detecting differential sgRNAs and genes targeted by sgRNAs. We followed authors’ tutorials (https://sourceforge.net/p/mageck/wiki/demo/#the-second-demo-starting-from-raw-fastq-files) to perform MAGeCK on RT112 and UMUC3 CRISPR screen data. At gene level, we compared sgRSEA, ScreenBEAM^63^, PBNPA^17^ and MAGeCK to NBBt-test using the CRISPR screening datasets of RT112 and UMUC3. The sgRSEA, ScreenBEAM and PBNPA were implemented by following their R packages. For differential splicing event analysis, many methods or tools, for example, DEXseq^26^, DSGseq^64^, SplicingCompass^33^, rMATS^34,35^, Cufflinks^37^, SeqGSEA, rdiff-parametrics^65^, and DiffSplice^38^) have been developed. However, since these methods are based on different models (such as count-based or isoform resolution-based), we chose DEXseq for comparison.

### F1-score

F_1_-score is a measure of a test's accuracy in statistics and widely used in the field of information retrieval for measuring search, document classification, and query classification performance^66^. F1-score is defined as

5 $$\begin{aligned} F1 & = \frac{1}{{\frac{1}{2}\left( {\frac{1}{recall} + \frac{1}{precision}} \right)}} = \frac{2}{{\frac{1}{recall} + \frac{1}{precision}}} \\ & = \frac{2}{{\frac{1}{recall} + \frac{1}{precision}}}\frac{recall \times precision}{{recall \times precision}} \\ & = 2\frac{recall \times precision}{{recall + precision}} = \frac{{2\left( {\frac{tp}{{tp + fn}}} \right)\left( {\frac{tp}{{tp + fp}}} \right)}}{{\frac{tp}{{tp + fn}} + \frac{tp}{{tp + fp}}}} = \frac{2tp}{{2tp + fn + fp}} \\ \end{aligned}$$

where

6 $$\begin{aligned} recall & = \frac{true\;positives\;discovered\;by\;a\;method}{{total\;true\;positives\;given\;in\;a\;pupolation}} = \frac{tp}{{tp + fn}} \\ & \quad \quad \left( {0 \le recall \le 1} \right) \\ \end{aligned}$$

7 $$\begin{aligned} precision & = \frac{true\;positives\;discovered\;by\;a\;method}{{total\;findings}} = \frac{tp}{{tp + fp}} \\ & = \frac{total\;findings - false\;positivs}{{total\;findings}} \\ & = \frac{total\;findings}{{total\;findings}} - \frac{false\;positivs}{{total\;findings}} = 1 - FDR \\ & \quad \quad \left( {0 \le precision \le 1} \right). \\ \end{aligned}$$

where $tp$ = true positives, $fn$ = false negatives, and $fp$ = false positives. F1-score is a harmonic mean of the precision and recall. F1-score reflects balance between the precision and the recall of the tests and hence also reaches its best value at 1 (perfect precision and recall) when all true positives are found or $fn$ = 0 and $fp$ = 0.

### N-score

N-score: Many heatmaps were made by using z-score: a standard normal value: $z=\frac{x-\overline{x}}{\sigma }$ where $\overline{x }$ and $\sigma$ are respectively average expression value and standard deviation of a gene over all replicates. z-score separates data into two groups: positive and negative and are in range of − 4 to 4 or less for expression of all genes, so it can visualize difference in expression of genes in two conditions. However, it just is because all data are separated into positive and negative groups, heatmap can clearly display difference in expression between two conditions even though the difference in expression of some genes between two conditions are not enough distinct. Therefore, z-score heatmap may show us false difference between two conditions. To avoid this false difference visualization, we here gave n-score for heatmap: Let expression value(x) of a gene or an isoform be log2 transformation value, we then use y = 2^×^ to re-convert into the original expression value (y) and use ${n}_{ij}={y}_{ij}\frac{\max(y)}{\max({y}_{i})}$ to build n-score where max(y) is maximum among all genes and all replicates of two groups and $\max({y}_{i})$ is maximum among all replicates of two groups in gene i. $n$ has the same order of magnitude for all genes detected in high-throughput experiment but it does not have positive or negative value. Therefore, unlike z-score, n-score heatmap cannot visualize distinct difference in expression of genes between two conditions if the difference between conditions is not at an order level.

### NBBt-test package

NBBt-test can be implemented by R package, NBBttest. Package NBBt-test was recruited by CRAN and can be installed into R console (Mac) or RGui (PC) or Rstudio. In addition, NBBttest can also be downloaded from github (https://github.com/Yuande/NBBttest) where README.md provides a detail instruction for user to run NBBt-test. Using NBBt-test one can check quality of count data, do multiple differential analyses of alternative splicing, alternative polyadenylation, CRISPR knockout screening, or gene expression, visualize the results and use NBBplot to display differential expression of exons within a specified DE gene identified by NBBt-test between two conditions across all replicates. NBBt-test package has two heatmap functions, myheatmap and myheatmap2. Function myheatmap uses row z-score across the sample individuals to show differential expression of genes or isoforms selected by NBBt-test between two conditions in one or multiple datasets. Function myheatmap2 uses n-score to show differential expression of genes or isoforms selected by NBBt-test between two conditions in one or multiple datasets.

## Supplementary Information

Supplementary Information 1.

Supplementary Figures.

## Data Availability

Source data used in this study are publicly available from corresponding original publications as follows: Pasilla RNA-seq count data from Brooks et al.^53^ with Gene Expression Omnibus (GEO) accession numbers, GSM461176–GSM461181; CRISPR knockout screen data was downloaded from Evers et al.^55^ with Short Read Archive (SRA) accession code, SRP072947; Poly(A) RNA-seq count data and qPCR data from Tan et al.^42^; and the *Arabidopsis* heat shock RNA-seq data from Gulledge et al.^50^ accessible at https://www.arabidopsis.org/servlets/TairObject?type=gene&name=AT1G07350.1.

## Code availability

The source code for NBBt-test is accessible as an R-package download from R CRAN (https://CRAN.R-project.org/package=NBBttest) and  https://github.com/Yuande/NBBttest.
