# Supplementary material for: NBBt-test: a versatile method for differential analysis of multiple types of RNA-seq data
Source: Sci Rep. 2022 Jul 27;12:12833. doi: 10.1038/s41598-022-15762-x (PMC9329447; doi:10.1038/s41598-022-15762-x)
Supplement: Supplementary file 1 — Supplementary Information 1. [file 41598_2022_15762_MOESM1_ESM.docx]

**Statistical Methods**

**Parameter estimations**

For the sake of convenience, we begin with sgRNA count data. Suppose we collect G genes of interest and gene g (g = 1, …, *G*) has $n_{g}$sgRNAs in a screening experiment. Let $x_{gij}$be a normalized or adjusted read count of sgRNA *i* (*i*=1, ..., $n_{g})$ targeting gene g in experiment (biological replicate experiment) *j* (*j* =1, …, *m*)*.* For the connivance, our method is restricted to sgRNAs targeting a gene, in other words, count data of multiple sgRNAs within a gene are used as a data matrix. Our method first works in this matrix and iteration with G matrices. The count of sgRNA reads is assumed to follow negative binomial distribution^1^:

$x\sim NB(r,p)$ (S1)

where $r$is a specified number of failures to sequence RNA species and *p* is probability of this RNA species (also called RNA isoform) that is sequenced. For the count data of CRIPSR knockout screen, *p* is estimated by proportion of sequencing RNA sequences from gene *g* targeted by a sgRNA*.* For the convenience, we refer to the proportion as proportion of the sgRNA targeting the gene. We here use

$p_{gij}=x_{gij}/X_{g}$ (S2)

to estimate proportion of sgRNA *i* within gene *g* in experiment *j* where $X_{g}$is the largest total count over all $n_{g}$ sgRNAs among $m$ replicate experiments. Using $X_{g}$instead of $X_{gj}$to calculate$p_{gij}$is because a set of sgRNAs are already designed before experiment and hence difference among replicate experiments is due to technical noise instead of biological system error. Using $X_{gj}$to calculate $p_{gij}$would increase proportion of technical noise due to the fact that $X_{gj}$contains noise anong m replicates. Our simulation also shows that $p_{gij}=\frac{x_{gij}}{X_{g}}$ is better than $p_{gij}=\frac{x_{gij}}{X_{gj}}$ (results not shown). Although $\sum_{i}^{n_{g}} p_{gij}\approx1$,$p_{gij}$is still assumed to follow a beta distribution^2^:

$p\sim Beta(\alpha,\beta)$. (S3)

Mean and variance of the proportion for a sgRNA targeting a gene are given by $\alpha$ and $\beta$^3^ :

$E(p)=\frac{\alpha}{\alpha+\beta}$ , (S4)

$V(p)=\frac{\alpha\beta}{(\alpha+\beta)^{2}(\alpha+\beta+1)}$ . (S5)

To avoid confusion, we denote $a$and $\beta$as $\alpha_{gi}$and $\beta_{gi}$for beta parameters of proportion of sgRNA *i* targeting gene *g*. To consider the case that CRISPR knockout screening experiment replicates are limited, we use weights to correct biases of variance of the estimate of $p_{gij}$ across *m* replicate experiments^3,4^:

(S6)

(*i*=1, …, *n*_g_; *j*=1, …, *m*; *g* =1, …, *G*).

(S7)

(*i*=1, …, *n*_g_; *j*=1, …, *m*; *g* =1,…, *G*)

where $\sum_{j=1}^{m} w_{gj}=1$ is constrained. Equation (S6) indicates that this combination has the expectation of mean. With the weights, the proportion of sgRNA *i* targeting gene *g* in a condition is estimated by

, (*i* =1, …, *n*_g_; j=1, …, *m*; *g* =1, …, *G*), (S8)

and the variance is also estimated in an unbiased fashion^3,4^:

, (*i*=1, …, *n*_g_; *j*=1, …, *m*; *g*=1, …, *G*). (S9)

Equation (S9) is derived from

(S10)

where

$W=\sum_{j=1}^{m} w_{j}=1$and $W^{2}=\sum_{j=1}^{m} w_{j}^{2}$, (S11)

$E\left[X\right]=E[\sum_{j}^{m} w_{j}x_{j}]=\sum_{j}^{m} w_{j}E\left[x\right]=\mu$, (S12)

$V\left(X\right)=V\left(\sum_{j=1}^{m} w_{j}x_{j}\right)=\sum_{j=1}^{m} w_{j}^{2}V\left(x_{j}\right)=\sum_{j=1}^{m} w_{j}^{2}\sigma^{2}$ . (S13)

Weight $w_{j}$in Equations (S6-S9) can be solved using Lagrange multipliers:

(S14)

For poly(A) RNA-seq or splicing/exon RNA-seq, since RNA in an experiment is abstracted from a library, different libraries would have different total RNA amounts, that is, RNA amount is fixed by library size. To remove difference due to library sizes, $p_{gij}$ in Equation (S2) is defined as

$p_{gij}=\frac{x_{gij}}{X_{gj}}$ (S15)

where $X_{gj}=\sum_{i=1}^{n_{g}} x_{gij}$.

**Iteration algorithm for optimal estimation of parameters**

Since we have weights for parameters ($\alpha,\beta$, $p$, and *V* ) in a given condition, then an iteration algorithm for optimal estimation of these parameters can be driven by estimating weights^3,4^.

At the initial step, we set

$w_{gj}^{(0)}=\frac{X_{gj}}{\sum_{j=1}^{m} X_{gj}}$, $\hat{p}_{gij}=\frac{x_{gij}}{X_{g}}$, (S16)

(S17)

At step k, we have

, (S18)

, (S19)

$w_{gj}^{(k)}=\frac{\left(\alpha_{g}^{(k-1)}+\beta_{g}^{(k-1)}\right)X_{gj}}{\alpha^{(k-1)}+\beta_{g}^{(k-1)}+X_{gj}}$, (S20)

, (S21)

. (S22)

$d_{gj}^{(k)}=w_{gj}^{(k)}-w_{gj}^{(k-1)}$ (S23)

Step *k* is looped by iteration. At the final step, the iteration stops and exits out of loop when

$d_{gj}^{(k)}\leq\Delta$ (S24)

where $\Delta$is a given tolerant threshold. We use final estimates of variance and proportion as their estimates: and $\hat{p}_{gi}=p_{gi}^{(k)}$.

In all RNA-seq data many RNA isoforms or sgRNAs have small read counts. Small counts would result in extremely similarity of proportions among few replicates, which leads variances to be much smaller than differences between means so that the t-statistics are inflated^4^. To avoid occurrence of this phenomenon, we propose another alternative estimate of *p* variance:

$\hat{V}_{gi}^{\#}=\frac{1}{X_{g}}\left[\frac{1+X_{gi}}{X_{g}}\left(1-\frac{1+X_{gi}}{X_{g}}\right)\right]$ (S25)

where $X_{gi}=\sum_{j=1}^{m} x_{gij}$and $X_{g}=\sum_{i=1}^{n_{g}} X_{gi}$. The variance for proportion (p) of sgRNA *i* targeting gene *g* or RNA isoform *i* within gene *g* is given by choosing bigger one from these two estimated variances:

. (S26)

Equation (S25) shows that the lower bound of $\hat{V}_{gi}^{\#}$is $\frac{1}{X_{g}^{2}}\left( 1-\frac{1}{X_{g}} \right)$> 0 when $\sum_{j=1}^{m} x_{gij}=0$ and when$\sum_{j=1}^{m} x_{gij}$is extremely small. Thus when $\hat{V}_{gi}^{*}<V_{gi}^{\#}$.

**t-test for differential expressions of RNA isoforms or sgRNAs**

With given$\hat{p}_{Agi}$ and $\hat{p}_{Bgi}$, $\hat{V}_{Agi}$and $\hat{V}_{Bgi}$in conditions A and B, a t-like statistic^4^ (we call the t-like statistic because it is not but very similar to standard t-test and we also call it t-test for the convenience) for differential expression of isoforms or hit of sgRNA *i* targeting gene *g* is defined as

(S27)

($\alpha$ is significance level,$i=1,\cdots,n_{g}$and $g=1,\cdots,G$),

and degree of freedom(df) is

(S28)

where

(S29)

$\bar{X}_{gki}=\frac{1}{m_{k}}\sum_{j=1}^{m_{k}} x_{gkij}$ (k=1 for A and 2 for B) (S30)

Although CRISPR sgRNAs and RNA isoforms occur at sub-gene level, a set of CRISPR sgRNAs targeting DNA sequence of a gene was already designed before experiment, the variation of sgRNAs targeting reads is fixed within a gene or due to a fixed effect while sgRNA target variation at gene level is uncertain, depends on gene structure and function, in other words, due to random effect. This character has not been considered in all current existing methods. To use information of this character, we introduce gene-wise $\rho$ to adjust difference of sgRNA counts targeting a gene between conditions A and B in Equation (S27) and use total counts of a gene to calculate degree of freedom of each of a sgRNA set in Equation (S28). RNA isoforms are derived from alternative splicing sites or alternative polyadenylation sites, hence, amounts of RNA isoforms are uncertain at sub-gene and gene levels, or depend on response of the gene to condition effect, that is to say, variation of a RNA isoform is not fixed at either splicing or poly(A) sites and at gene level but due to random effect. For this reason, we introduce isoform-wise $\rho$ to adjust difference between two conditions in Equation (S27) and also use isoform-wise mean to calculate degree of freedom in Equation (S28). In addition, we use $a_{gi}$ defined in Equation (S29) to adjust difference of degree of freedom (df) among isoforms or sgRNAs. The $a_{gi}$ is very useful, for example, difference between$\bar{X}_{gAi}$ and$\bar{X}_{gBi}$ is small, then $a_{gi}$ is very small, which leads df to be also small and p-value is large, otherwise, p-value is small.

We used $\rho$ to measure effects of small sample size and introduced it into t-test in Equation (S27) where$\rho_{gi}$is defined as geometric mean of $\phi_{gi}$ and $\zeta_{gi}$, $\rho_{gi}=\sqrt{\zeta_{gi}\phi_{gi}}$. To avoid zero count, we modify $\phi_{gi}$as

$\phi_{gi}=\max\left[\frac{\min(X_{gAi})+1}{\max(X_{gBi})+1},\frac{\min(X_{gBi})+1}{\max(X_{gAi})+1}\right]$ (S31)

and $\zeta_{gi}$ also is modified as

(S32)

$\phi_{gi}$ is used to measure overlap between datasets A and B of sgRNA *i* targeting gene g or isoform *i* within gene *g* and $\zeta_{gi}$is used to measure homogeneity of data within conditions where $\bar{X}_{gAi}=\frac{1}{m_{A}}\sum_{j=1}^{m_{A}} x_{gAij}$and $\bar{X}_{gBi}=\frac{1}{m_{B}}\sum_{j=1}^{m_{B}} x_{gBij}$. For read count data, $x_{gAij}\geq0$ and $x_{gBij}\geq0$. If , then$\bar{X}_{gi}=0$ and $\zeta_{gi}=0$. Domains of $\phi_{gi}$and $\zeta_{gi}$are $0\leq\phi_{gi}\leq\infty$ and $0\leq\zeta_{gi}\leq\infty$, respectively.

**t-test for differential expression of genes**

Since selection may be biased^1^ or sgRNAs may have amount difference in CRISPR knockout screen experiments, which result in the fact that in the same genes, some sgRNAs become major sgRNA, while the others become minor sgRNAs. Such a difference among a set of sgRNAs would lead to difference in hitting on DNA sequence of a gene. However, this type of differences is not biological difference. To remove difference among sgRNAs, we need to consider information of genes targeted by sgRNAs. To this end, we consider mean or sum of counts over all designed sgRNAs targeting DNA sequence of a gene to represent hit count of this gene because mean and sum do not change variation of genes in a replicate. For the convenience, we here use sum

$X_{gj}=\sum_{i=1}^{n_{g}} x_{gij}$ (S33)

to represent hit count of a gene targeted by a set of sgRNAs. Similarly, $p_{gj}$is defined as proportion of gene *g* in replicate experiment *j* to the maximum total hit count across all genes among $m_{k}$:

$p_{gj}=\frac{X_{gj}}{\max(X_{j})}$ ($j=1, 2, \ldots, m_{k}, g=1,2,\ldots, G$), (S34)

$X_{j}=\sum_{g=1}^{G} X_{gj}$ ($j=1, 2, \ldots, m_{k}$). (S35)

where *k* is condition k, *k* =1 for A and 2 for B. Differential analysis of a set of G genes is similar to that of a set of sgRNAs targeting a gene. For Poly(A) isoform data, the $p_{gj}$ is defined as

(S36)

where $\bar{X}_{gj}=\frac{1}{n_{g}}X_{gj}$and $\bar{X}_{j}=\frac{1}{m_{k}}X_{j}$. For RNA-seq data without isoforms, the $p_{gj}$ is defined as

$p_{gj}=\frac{X_{gj}}{X_{j}}$ (S37)

We still suppose that $p_{gj}$ follows beta distribution. Therefore, we use Equations (S6-S24) by removing subscript *i* to estimate$p_{g}$and $V_{g}$in a condition. With $\hat{p}_{Ag}$and $\hat{P}_{Bg}$, $\hat{V}_{Ag}$and $\hat{V}_{Bg}$in conditions A and B, t-statistic for differential expression of gene g is defined as

($g=1,\cdots,G$) (S38)

with

(S39)

where

S40)

$m_{A}\mathrm{and} m_{B}$ are replicate numbers in conditions A and B, respectively.

References

^1^ Li, W. *et al.*, MAGeCK enables robust identification of essential genes from genome-scale CRISPR/Cas9 knockout screens. *Genome Biol* 15 (12), 554 (2014).

^2^ Casella, G. & Berger, R.L., *Statistical Inference* 2nd ed. (Wadsworth Group, Duxbury, 2002).

^3^ Baggerly, K.A., Deng, L., Morris, J.S., & Aldaz, C.M., Differential expression in SAGE: accounting for normal between-library variation. *Bioinformatics* 19 (12), 1477-1483 (2003).

^4^ Tan, Y.D., Chandler, A.M., Chaudhury, A., & Neilson, J.R., A powerful statistical approach for large-scale differential transcription analysis. *PLoS One* 10 (4), e0123658 (2015).
