# Supplementary material for: NBBt-test: a versatile method for differential analysis of multiple types of RNA-seq data
Source: Sci Rep. 2022 Jul 27;12:12833. doi: 10.1038/s41598-022-15762-x (PMC9329447; doi:10.1038/s41598-022-15762-x)
Supplement: Supplementary file 2 — Supplementary Figures. [file 41598_2022_15762_MOESM2_ESM.docx]

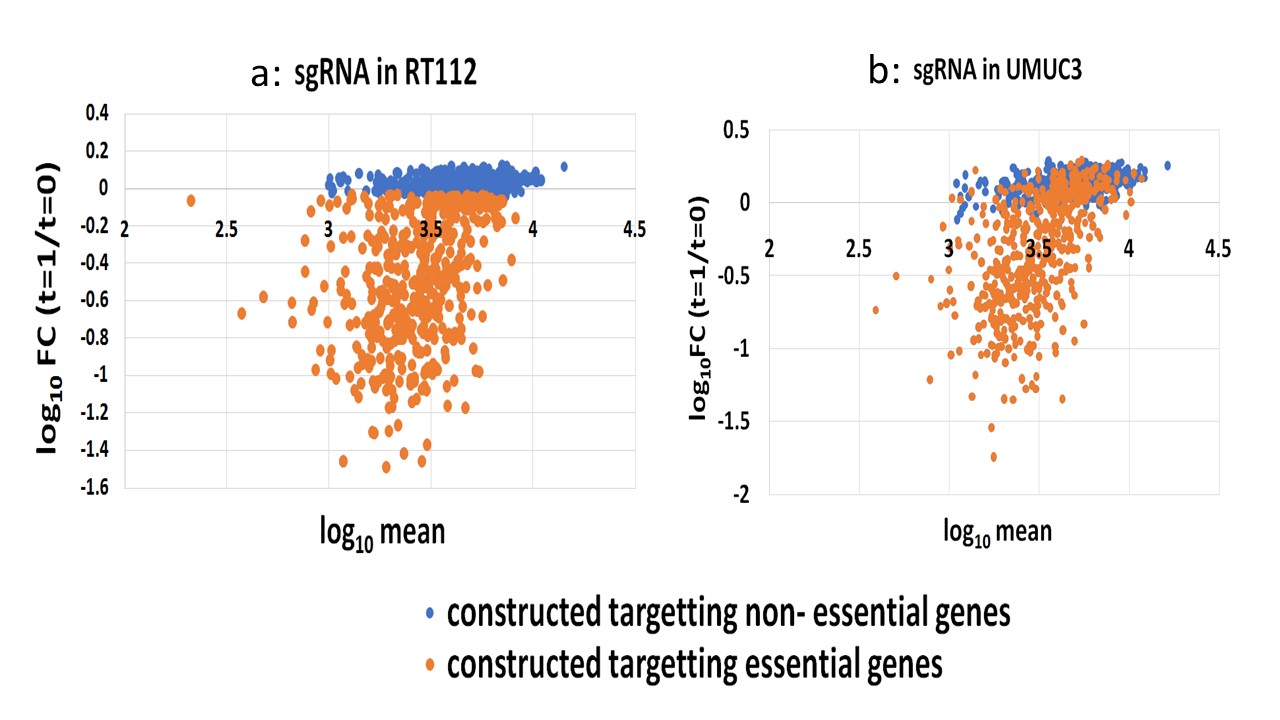


**Figure S1. MAplots of sgRNA screen data from cell lines RT112 and UMUC3.**

MAplot is a plot of log10(fold change(T1/T0)) vs log10(average over all replicates). The brown dots represent essential sgRNAs and blue dots denote the unessential sgRNAs. Left and right figures are MAplots of CRISPR knockout screening spike-in data from cell lines RT112 (A) and UMUC3(B), respectively. In RT112, essential and unessential sgRNAs are clearly separated at log10(fold change(T1/T0))=0 but in UMUC3, the unessential sgRNAs (blue dots) are mixed together with some of the essential sgRNAs (brown dots).


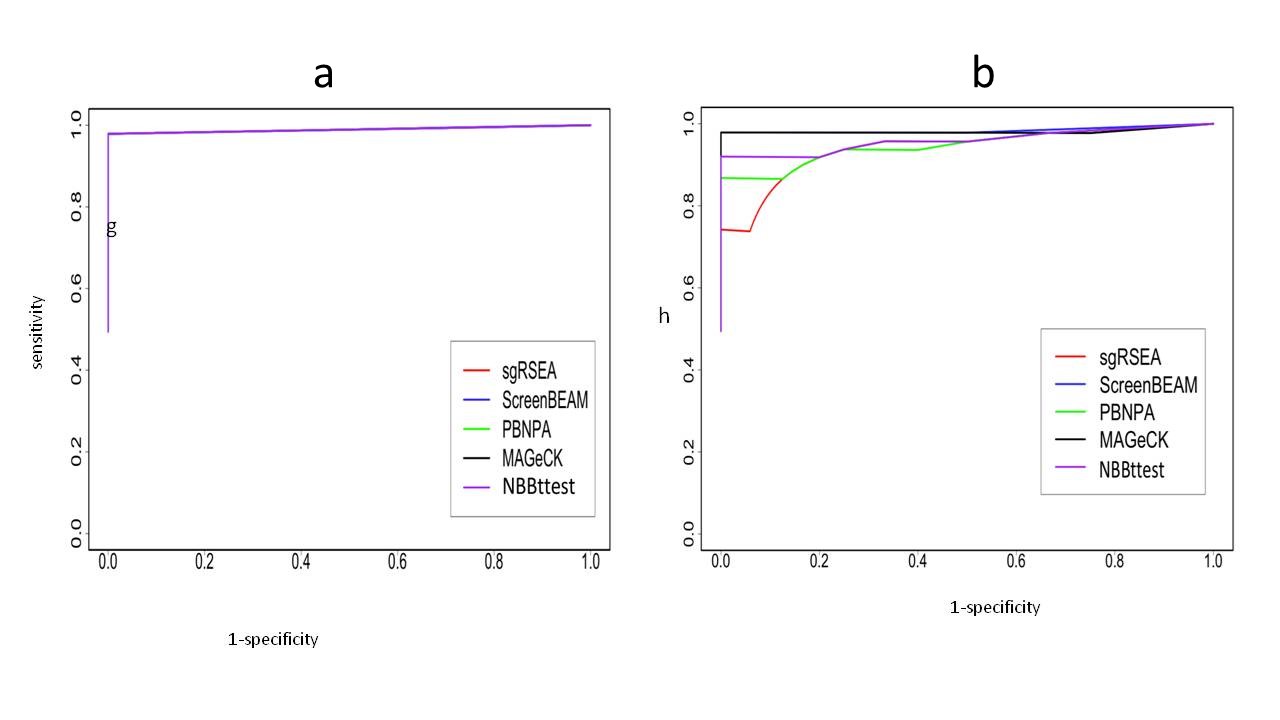


**Figure S2.** **Performance comparison among statistical methods in identifying differential CRISPR knockdown screened genes targeted by sgRNAs**

The ROC curves display performances of the five statistical methods for identifying truly differentially screened genes targeted by sgRNAs between T1 and T0 using CRISPR spike-in data from cell lines, RT112 (A) and UMUC3 (B), respectively. As shown in Figure S1A, RT112 is good data so that all 5 selected methods show perfect performances. UMUC3 is bad data (Fig. S1B) where ROC curves show that NBBttest had the best performance of identifying differential knockdown screened gene among the other 4 methods except for ScreenBeam that had a perfect performance.


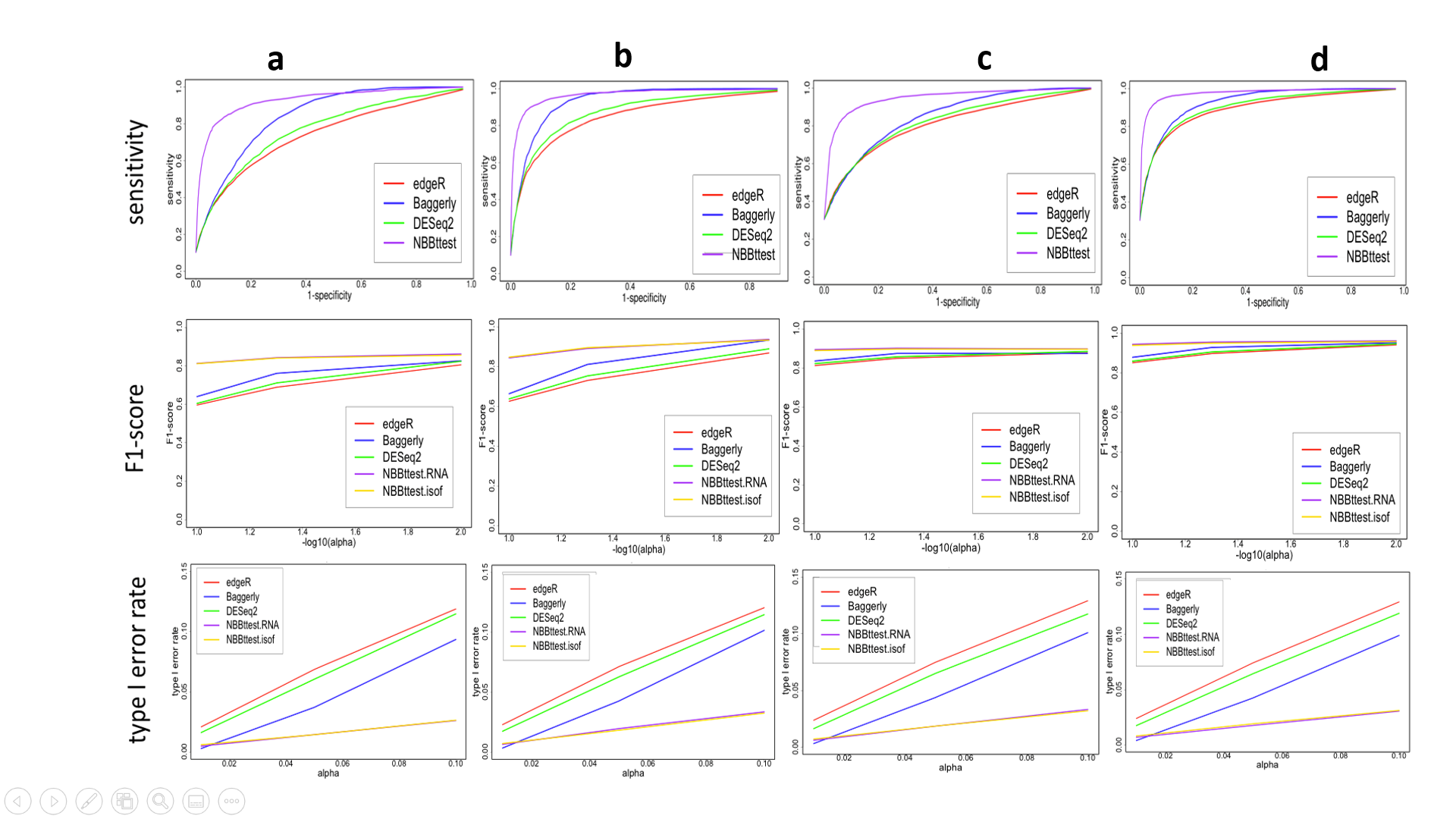


**
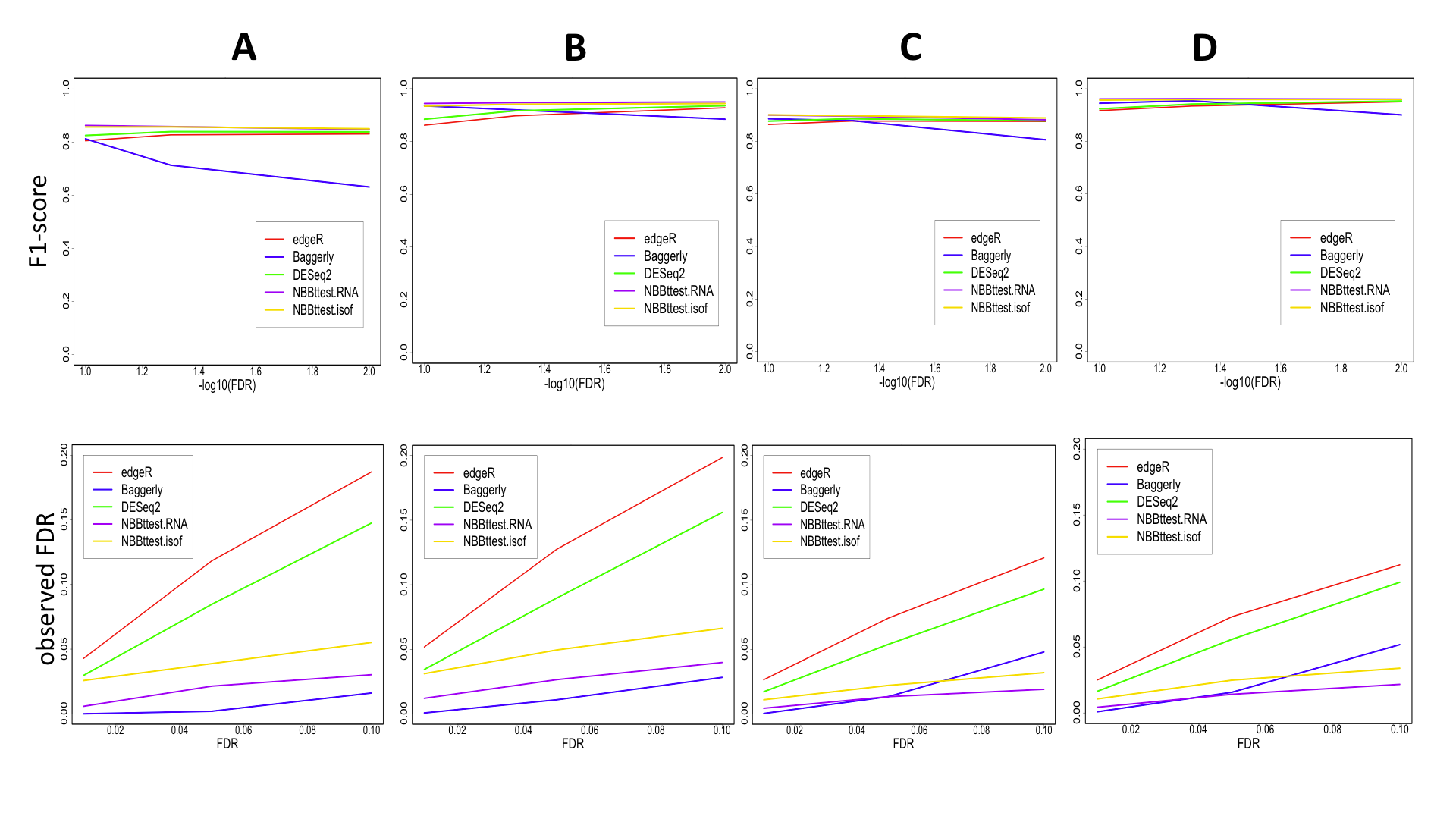
**

**Figure S3. Comparisons of performance, F1-score, and type I error rate, and observed FDRs of statistical methods in finding differential polyadenylation site**s

ROC curve, F1-score and type I error rate of a method were given by performing a statistical method on the simulated poly(A) count data. F1-score and type I error rate were calculated based on single-test results. The simulated poly(A) site count data were generated by using negative binomial distribution on the Jurkat T-cell poly(A) count data. We simulated four scenarios **a, b, c** and **d**. Each scenario had two samples with equal sample size of 5 and 13,409 poly(A) sites scattered in 9,294 genes and **30%** artificial noise (or outliers). **a:** T-cell stimulation effect: A=**100**U, where 0 < $U\leq1$ and **10%** of poly(A) sites were positively or negatively responded to anti-D3/anti-CD28 stimulation. **b:** T-cell stimulation effect: A=**300U** and **10%** poly(A) sites were positively or negatively responded to anti-D3/anti-CD28 stimulation. **c:** T-cell stimulation effect: A=**100U** and **30%** of poly(A) sites were positively or negatively responded to anti-D3/anti-CD28 stimulation. **d:** T-cell stimulation effect: A=**300U**; **30%** of poly(A) sites were positively or negatively responded to anti-D3/anti-CD28 stimulation.


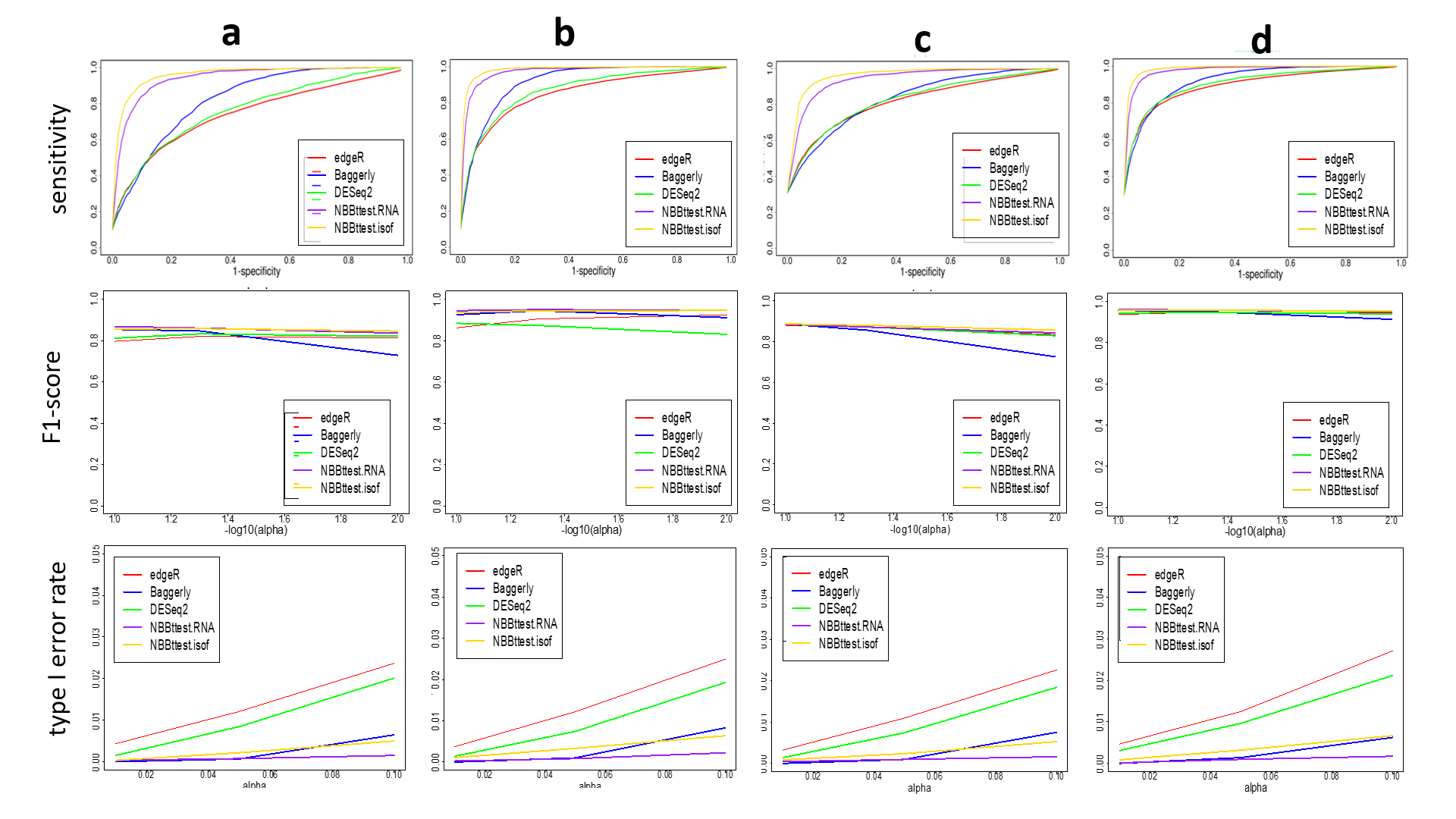


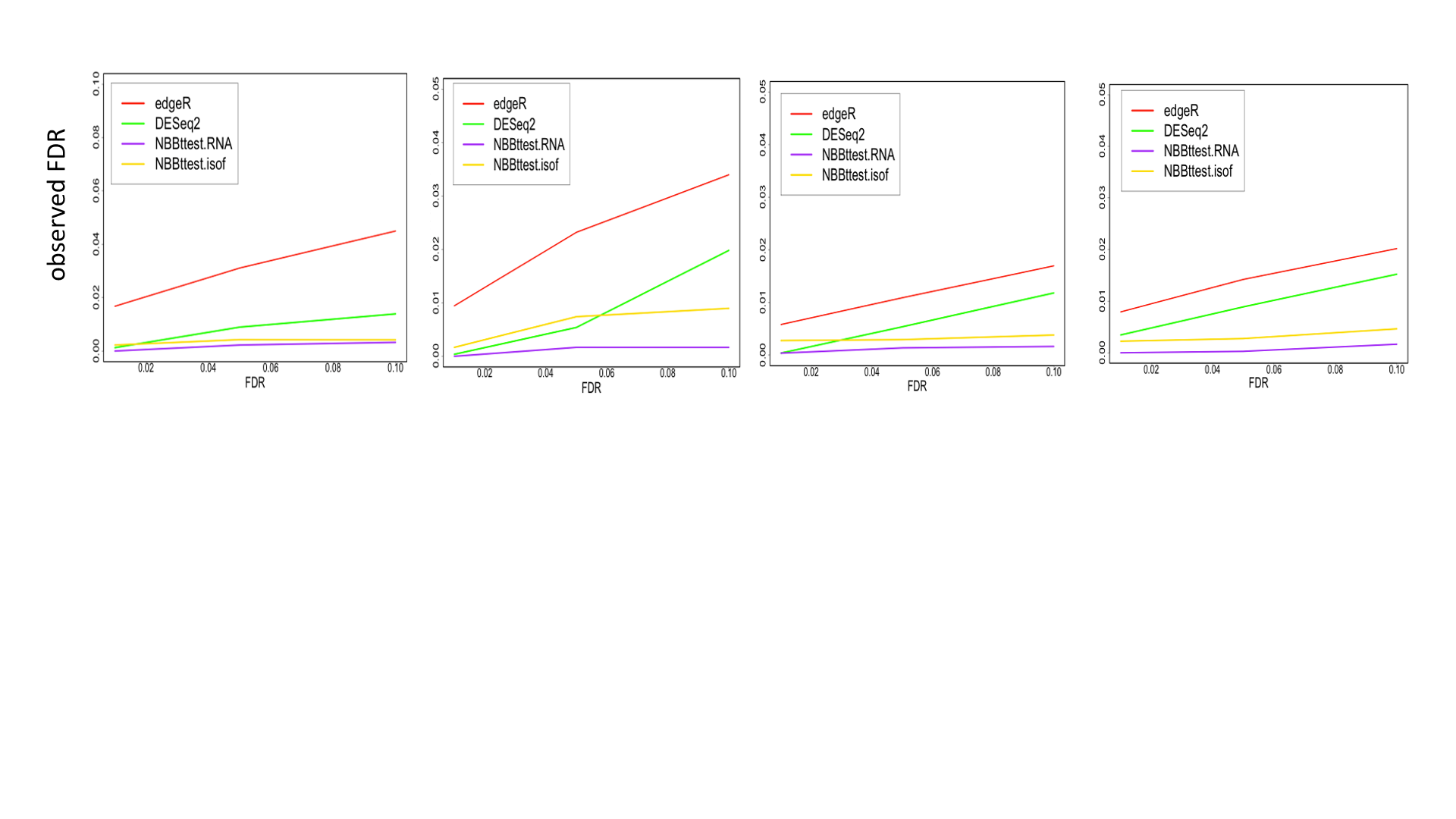


**Figure S4. Performance, F1-score, and type I error rate and observed FDR of statistical methods in finding differential polyadenylation sites**

ROC curve, F1-score and type I error rate of a method were calculated by performing a statistical method on the simulated poly(A) count data. F1-score and type I error rate were calculated based on single-test results. The simulated poly(A) site count data were generated by using negative binomial distribution on the Jurkat T-cell poly(A) count data. We simulated four scenarios A, B, C and D. Each scenario had two samples with equal sample size of 3 and 13409 poly(A) sites scattered in 9294 genes and **no** artificial noise(or no outliers). **A:** T-cell stimulation effect: A=**100U** where 0 < $U\leq1$; **10%** of poly(A) sites were positively or negatively responded to anti-D3/anti-CD28 stimulation. **B:** T-cell stimulation effect: A=**300U**; **10%** of poly(A) sites were positively or negatively responded to anti-D3/anti-CD28 stimulation. **C:** A=**100U**; **30%** of poly(A) sites were positively or negatively responded to anti-D3/anti-CD28 stimulation. **D:** T-cell stimulation effect: A=**300U**; **30%** of poly(A) sites were positively or negatively responded to anti-D3/anti-CD28 stimulation.


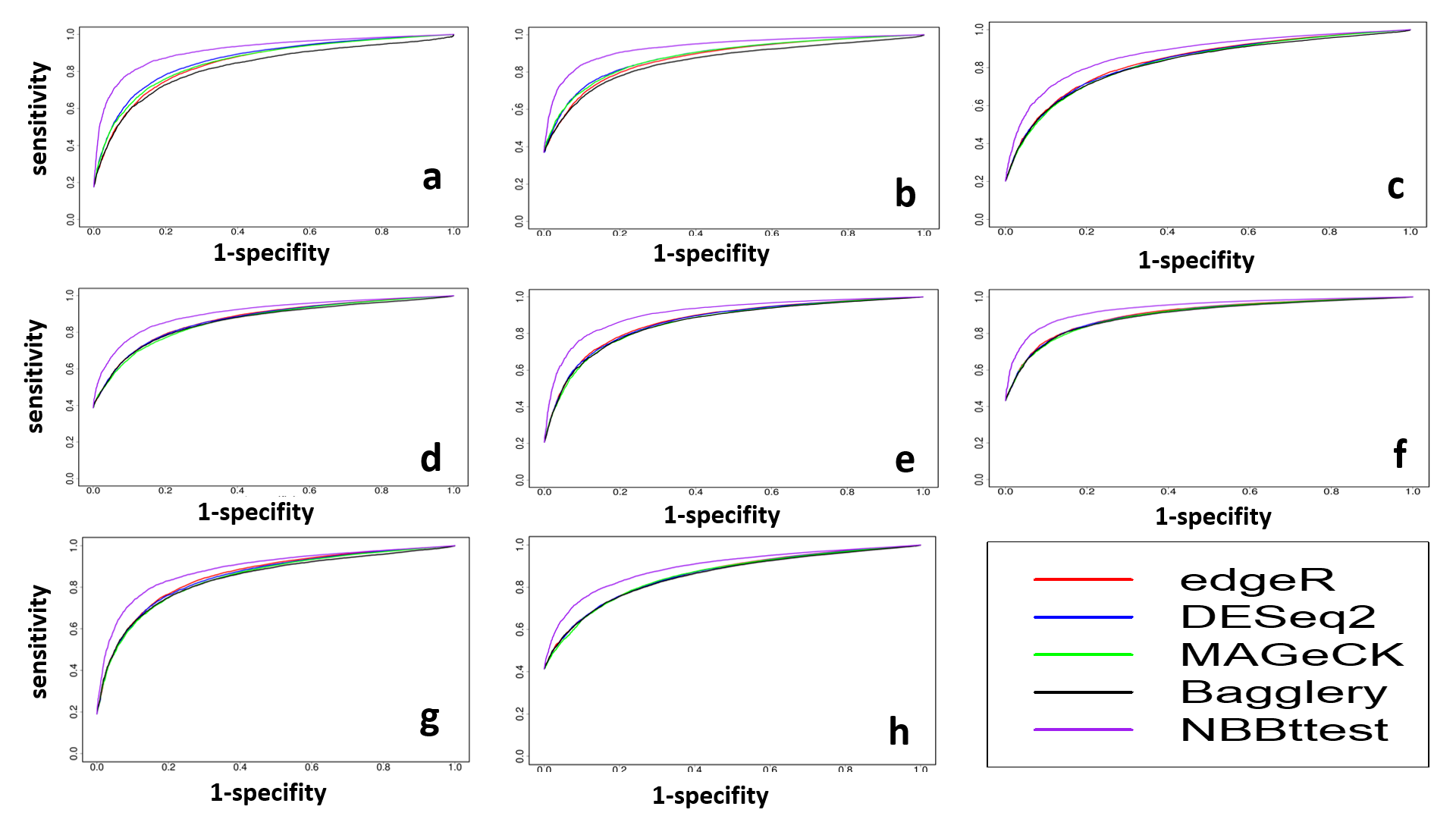


**Figure S5. Performances of statistical methods in finding differential CRISPR knockout screening sgRNAs**

Performances of statistical methods in identifying differential sgRNAs targeting genes in CRISPR knockout screening were analyzed by using the simulated data. The simulated FACS data of 1000 sets of sgRNAs, each set having 10 sgRNA targeting a gene, were generated by using CRISPulator. We simulated the following 8 CRISPR knockout screen scenarios, each having two samples with equal sample size of 4. Scenario **a:** FACS bin = 0.1, noise = 0.5, phenotype proportion = 0.2. Scenario **b:** FACS bin = 0.1, noise = 0.5, phenotype proportion = 0.4. Scenario **c:** FACS bin = 0.1, noise = 1.0, phenotype proportion = 0.2. Scenario **d:** FACS bin = 0.1, noise = 1.0, phenotype proportion = 0.4. Scenario **e:** FACS bin = 0.25, noise = 0.5, phenotype proportion = 0.2. Scenario **f:** FACS bin = 0.25, noise = 0.5, phenotype proportion = 0.4. Scenario **g:** FACS bin = 0.25, noise = 1.0, phenotype proportion = 0.2. Scenario **h:** FACS bin = 0.25, noise = 1.0, phenotype proportion = 0.4.


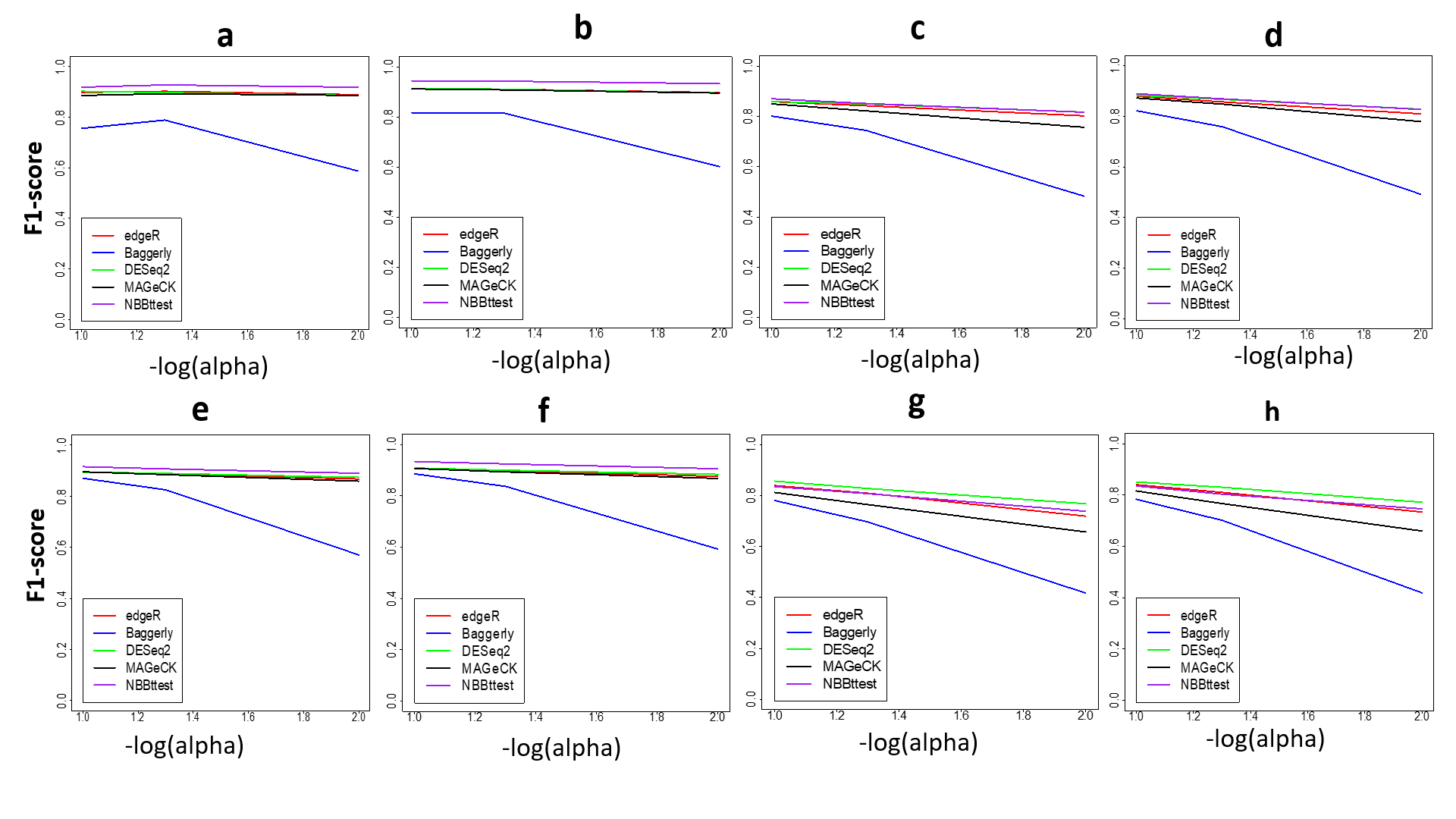


**Figure S6. F1-scores of statistical methods in finding differential sgRNAs targeting genes in CRISPR screen**

F1-score was calculated based on multiple-test results obtained by performing a statistical method on a simulated FACS dataset. The simulated FACS data were generated by using CRISPulator with CRISPR pooled screen. We simulated the following 8 CRISPR scenarios, each had two samples with equal sample size of 4: **a:** FACS bin = 0.1, noise = 0.5, phenotype proportion = 0.2. **b:** FACS bin = 0.1, noise = 0.5, phenotype proportion = 0.4. **c:** FACS bin = 0.1, noise = 1.0, phenotype proportion = 0.2. **d:** FACS bin = 0.1, noise = 1.0, phenotype proportion = 0.4. **e:** FACS bin = 0.25, noise = 0.5, phenotype proportion = 0.2. **f:** FACS bin = 0.25, noise = 0.5, phenotype proportion = 0.4. **g:** FACS bin = 0.25, noise = 1.0, phenotype proportion = 0.2. **h:** FACS bin = 0.25, noise = 1.0, phenotype proportion = 0.4.


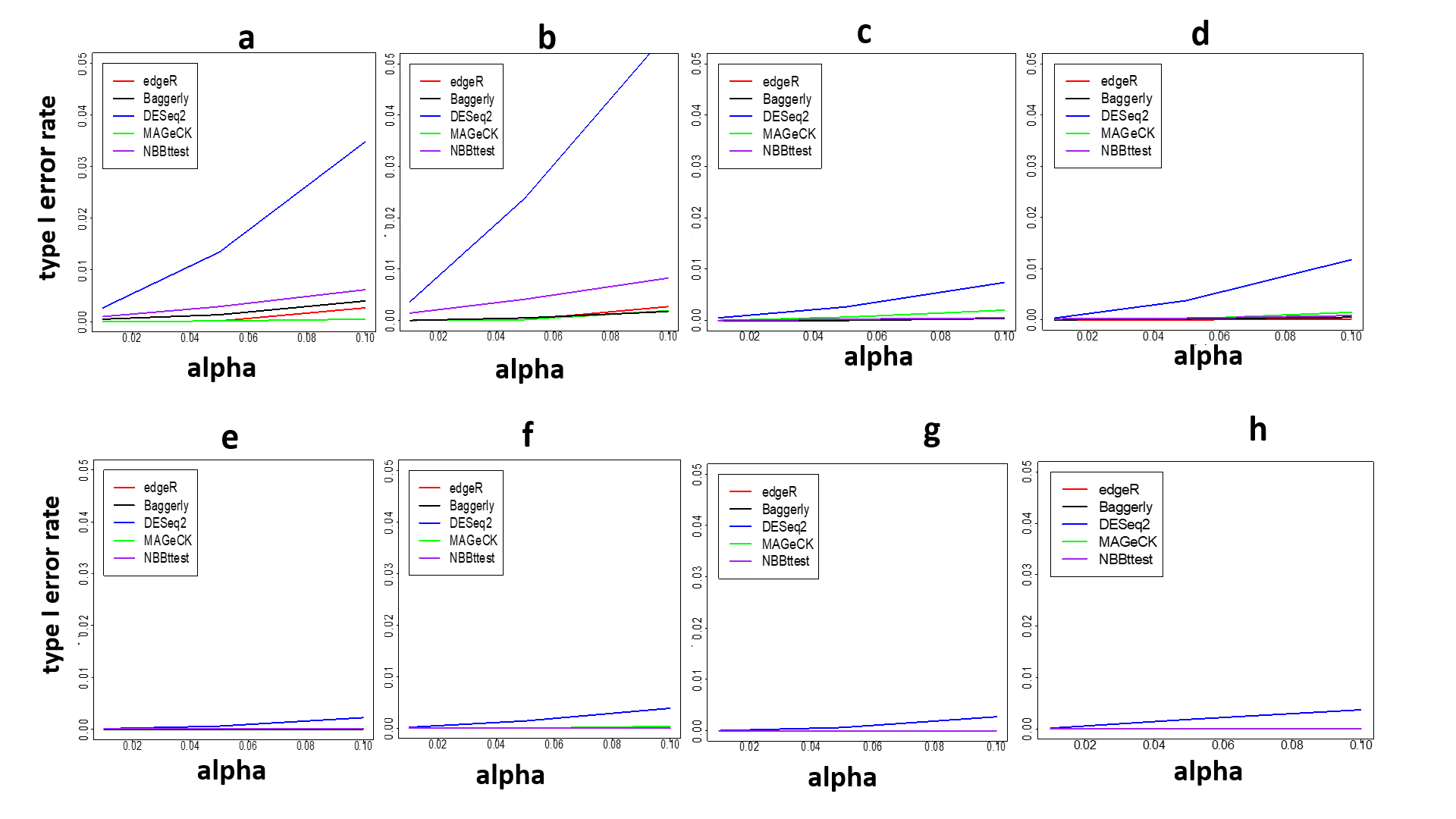


**Figure S7. Type I error rates of statistical methods in finding differential CRISPR screen sgRNA targets**

Type I error rate of a statistical method was calculated with the simulated FACS data. The simulated data were generated by using CRISPulator with CRISPR pooled screen. We simulated the following 8 CRISPR scenarios, each had two samples with equal sample size of 4: **a:** FACS bin = 0.1, noise = 0.5, phenotype proportion = 0.2. **b:** FACS bin = 0.1, noise = 0.5, phenotype proportion = 0.4. **c:** FACS bin = 0.1, noise = 1.0, phenotype proportion = 0.2. **d:** FACS bin = 0.1, noise = 1.0, phenotype proportion = 0.4. **e:** FACS bin = 0.25, noise = 0.5, phenotype proportion = 0.2. **f:** FACS bin = 0.25, noise = 0.5, phenotype proportion = 0.4. **g:** FACS bin = 0.25, noise = 1.0, phenotype proportion = 0.2. **h:** FACS bin = 0.25, noise = 1.0, phenotype proportion = 0.4.


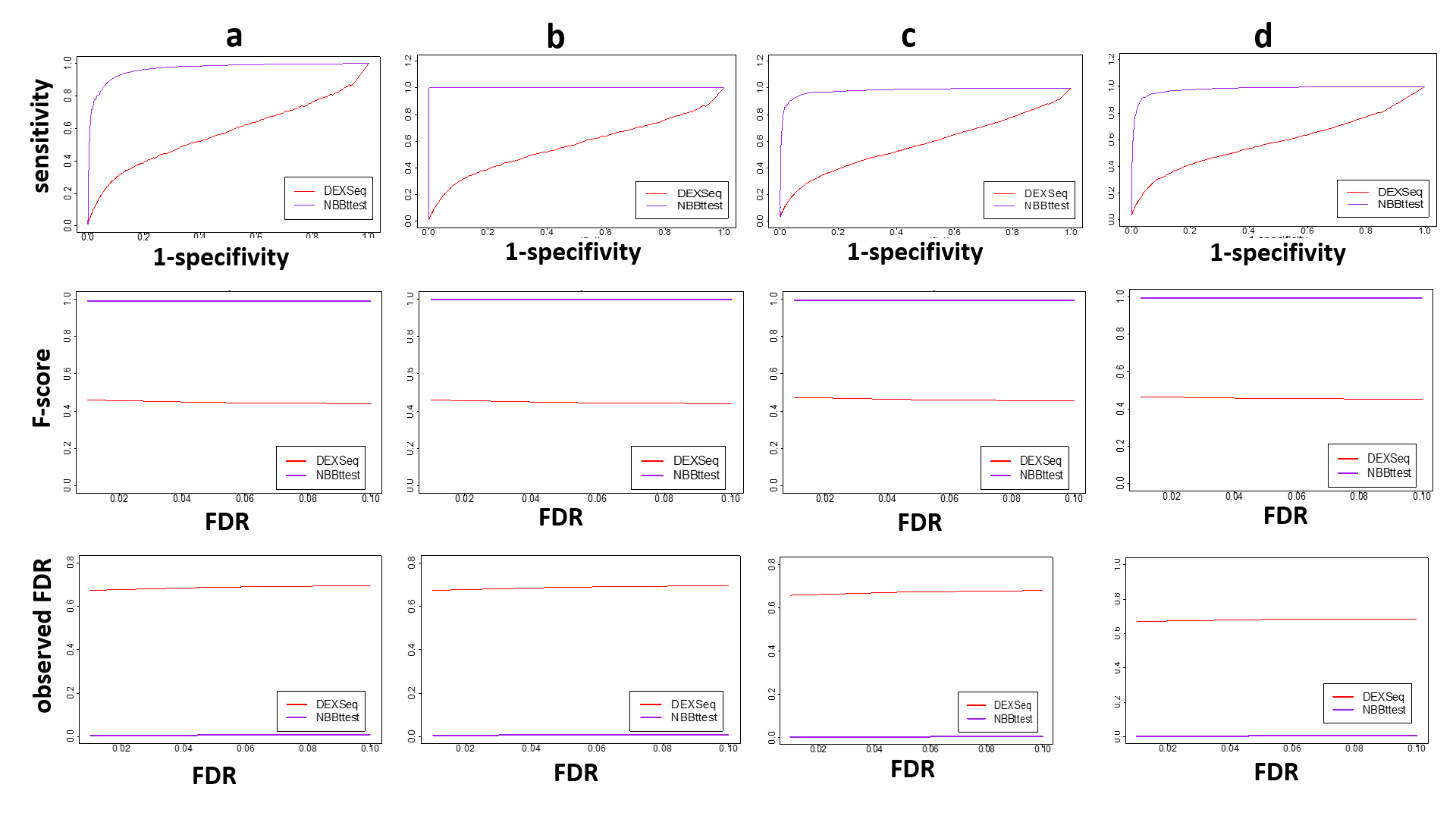


**Figure S8. Performance, F1-score and observed FDR of DEXSeq and NBBttest in finding differential isoforms due to alternative splicing**

The simulated splicing count datasets were generated from negative binomial distribution based on the pasilla exon splicing count data which have two samples and 69733 exons of 14206 Drosophila genes. Control sample has 4 biological replicates and knockdown sample has 3 biological replicates. We set four simulation scenarios.  **Scenario a:** knockdown effect A=**100U** where 0 < U $\leq1$, **10%** of exons were differentially spliced. **Scenario b:** knockdown effect A=**100U**, **30%** of exons were differentially spliced. **Scenario c:** Knockdown effect A=**300U**, 10% of exons were differentially spliced. **Scenario d:** Knockdown effect A=**300U**, **30%** of exons were differentially spliced.
